# Trapping gases in metal-organic frameworks with a selective surface molecular barrier layer

**DOI:** 10.1038/ncomms13871

**Published:** 2016-12-13

**Authors:** Kui Tan, Sebastian Zuluaga, Erika Fuentes, Eric C. Mattson, Jean-François Veyan, Hao Wang, Jing Li, Timo Thonhauser, Yves J. Chabal

**Affiliations:** 1 Department of Materials Science & Engineering, University of Texas at Dallas, Richardson, Texas 75080, USA; 2 Department of Physics, Wake Forest University, Winston-Salem, North Carolina 27109, USA; 3 Department of Chemistry and Chemical Biology, Rutgers University, Piscataway, New Jersey 08854, USA; 4 Department of Chemistry, Massachusetts Institute of Technology, Cambridge, Massachusetts 02139, USA

## Abstract

The main challenge for gas storage and separation in nanoporous materials is that many molecules of interest adsorb too weakly to be effectively retained. Instead of synthetically modifying the internal surface structure of the entire bulk—as is typically done to enhance adsorption—here we show that post exposure of a prototypical porous metal-organic framework to ethylenediamine can effectively retain a variety of weakly adsorbing molecules (for example, CO, CO_2_, SO_2_, C_2_H_4_, NO) inside the materials by forming a monolayer-thick cap at the external surface of microcrystals. Furthermore, this capping mechanism, based on hydrogen bonding as explained by *ab initio* modelling, opens the door for potential selectivity. For example, water molecules are shown to disrupt the hydrogen-bonded amine network and diffuse through the cap without hindrance and fully displace/release the retained small molecules out of the metal-organic framework at room temperature. These findings may provide alternative strategies for gas storage, delivery and separation.

## Abstract (web summary)

Metal-organic frameworks are extensively studied for gas storage applications, but one potential limitation is their relatively weak adsorption of gases. Here, the authors report that the exposure of metal-organic frameworks to ethylenediamine forms a monolayer thick cap which improves gas molecule retention.

Metal-organic framework (MOF) materials are crystalline nanoporous materials consisting of inorganic nodes (metal ions or clusters), also referred with secondary building units, and organic ligands as the connecting units^1^. Their high surface areas and micropore structure provide an ideal environment for adsorbing small molecules, which is the basis of many important applications such as energy storage and gas capture and separation^2^,^3^,^4^,^5^, and even biomedicine^6^. The main problem for gas storage is the relatively weak adsorption of many small gases in MOFs. The focus to enhance gas adsorption and separation has therefore been to develop metal centres that are more active (for example, exposed metal cations) and to functionalize the ligands by incorporating functional groups such as amine, hydroxyl and halide in the organic ligands to increase or tune the guest-host interaction^2^,^4^,^7^. These approaches tend to target specific molecules, for example, through the formation of Lewis acid–base pairs, and thus lack a wider applicability^4^. Furthermore, it requires novel and potentially complex synthesis procedures and often leads to a decrease of the internal surface area^7^,^8^,^9^.

An alternative approach is to find a way to cap the MOF microcrystals at the end of the loading process. While previous work has considered high molecular weight compounds such as polydimethysiloxane^10^ or copolymer Pluronic P-123 (ref. 11) to coat MOFs external surfaces by high temperature vapour-phase deposition or liquid immersion, these methods have not been used for and are not compatible with gas storage and release. Ethylenediamine (EDA) molecules, on the other hand, have low-molecular weight, relatively high vapour pressure (∼10 Torr, 20 °C) at ambient condition and contain terminal amine groups in EDA molecules, which are known to interact more strongly with a variety of MOFs, particularly those with open or unsaturated metal sites (for example, found in MOF-74) by forming metal-amine complexes^12^,^13^,^14^. Moreover, previous studies have shown that EDA molecules cannot easily penetrate into MOFs due to their size and strong interaction with the framework, requiring refluxing in solution (for example, anhydrous toluene)^12^,^15^,^16^. For instance, in Mg-MOF-74, that is, Mg_2_(dobdc) with dobdc=2,5-dihydroxybenzene dicarboxylic acid, the best attempts only lead to ∼0.13 EDA per Mg^2+^ metal centre, which is an order of magnitude less than theoretically possible^12^,^16^. The difficulty to fully load EDA in MOF-74 highlighted by these pioneering studies suggests that, without extensive refluxing, EDA molecules should only adsorb on the surface of MOF crystals. They are therefore attractive to coat external surfaces of MOFs, particularly MOF-74 that has a three-dimensional honeycomb lattice with one-dimensional (1D) channels (diameter ∼14 Å, Supplementary Fig. 1). In addition, MOF-74 contains a high density of coordinatively unsaturated metal sites, which are the highest binding energy sites for small molecules such as CO_2_, NO, SO_2_, CH_4_ and H_2_ (refs 17, 18, 19, 20, 21). Capping the end of the 1D channels, which constitute the only diffusion pathway for these small molecules^22^, could therefore be effective for storage of many such small molecules.

In this work, we demonstrate, using *in situ* infrared spectroscopy^23^,^24^,^25^ that is well-suited to determine absolute gas loading^25^, that post exposure of MOF-74 crystals to vapours of a ‘sticky' molecule such as EDA is very effective in trapping weakly bound small gas molecules (CO, CO_2_, SO_2_, C_2_H_4_) within the material, or to prevent their loading into an EDA-capped empty MOF. A combination of X-ray photoelectron spectroscopy with gas cluster ion sputtering (GCIS) and low-energy ion spectroscopy measurements establish that EDA is only adsorbed as a monolayer on the exterior surface of MOF crystals (<1 nm thick), that is, within the outermost pores of the microcrystals, thereby acting as capping molecules. *Ab initio* modelling provides an explanation for this observation and proposes a structure that accounts for the observed properties. Interestingly, this EDA barrier is transparent to water molecules that readily diffuse through it and remove pre-adsorbed molecules (for example, CO). *Ab initio* modelling attributes such a ‘gate opening' to the disruption of the H-bonded amine groups of EDA by water molecules.

## Results

### Characterization of EDA capping with CO molecules

We have initially focused on CO adsorption in Ni-MOF-74 because CO is a good probe of Lewis acid adsorption sites and sensitive to the local cationic environment^26^,^27^. Furthermore, the stretch frequencies (*ν*(CO)) of adsorbed and gas-phase species are easily distinguishable. Moreover, the CO-binding energy is higher in Ni-MOF-74 (∼52.7 kJ mol^−1^ determined by isotherm measurement^28^) than in other isostructural frameworks with *M*=Mg, Mn, Fe, Co and Zn (ref. 28). In all frameworks, the isotherms are fully reversible at room temperature, consistent with weak binding with uncoordinated metal sites through electrostatic, *σ* and *π* orbital interactions^28^.

**Figure 1:**
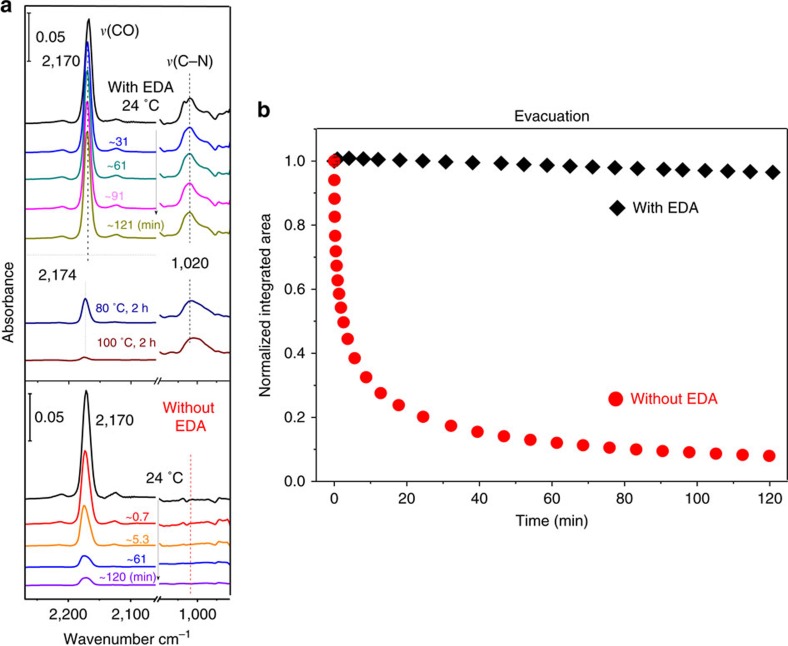
Time evolution of the *ν*(CO) band at 2,170 cm^−1^ upon evacuation. (**a**) Infrared spectra of adsorbed CO and EDA in pristine (bottom) and EDA post-loaded (top) Ni-MOF-74 sample upon evacuation (<20 mtorr). All spectra are collected at 24 °C and referenced to the pristine activated MOF in vacuum. The annealing sequence includes 2 h at 80 °C, cooling back to room temperature for data collection, and an additional 2 h at 100 °C, cooling back to room temperature for data collection. (**b**) Normalized integrated area of *ν*(CO) band at 2,170 cm^−1^ upon evacuation for pristine (red circles) and EDA post-loaded (black diamonds) Ni-MOF-74 sample. The integrated areas are normalized to their maximum value obtained at *t*=0 (top spectrum). The error bars are calculated from the variations in the measured (normalized) integrated areas and do not exceed 0.02.

After activation and CO loading (∼40 Torr), the *ν*(CO) band is first observed at 2,174 cm^−1^, then shifts to 2,170 cm^−1^ as saturation is reached (∼30 min)^28^,^29^; in both cases it remains clearly distinct from the gas-phase band centred at 2,143 cm^−1^ (Supplementary Fig. 2). The main shift is attributed to the formation of a Ni^2+…^CO adduct within the open metal site^29^. The coverage-dependent shift (∼−4 cm^−1^) is attributed to additional CO–CO lateral interaction and/or potential slight structural rearrangement of the metal-adduct as the loading increases^21^,^24^,^29^. The occupation reaches ∼0.7 molecules per metal site at ∼40 Torr^28^. Upon evacuation (pressure <20 mTorr), CO is removed within ∼30 min as shown in the red curve in Fig. 1.

If immediately upon evacuation (<∼3 s) a CO/EDA gas mixture (∼40 Torr/∼4 Torr) is introduced into the cell (when>∼95% CO is still trapped) and kept for ∼10 min, the intensity of the CO band remains constant (Supplementary Fig. 2). Furthermore, when the system is evacuated (pressure<20 mTorr), the CO band decreases by <3% after a 2-h evacuation, as shown in the black diamond of Fig. 1. These data unambiguously show that CO can be trapped by introduction of EDA vapour, without hindering the total MOF capacity for CO adsorption.

Detailed information about the nature of the EDA is obtained in the infrared absorption spectrum (Supplementary Note 1 and Supplementary Fig. 2): on one hand, the two strong *ν*_as,s_(–CH_2_) vibrational peaks at 2,936 and 2,860 cm^−1^ indicate that gas-phase EDA is clearly present in the CO/EDA mixture, although they quickly disappear as EDA molecules are adsorbed onto the MOFs and the cell internal walls. On the other hand, evidence for adsorbed EDA on or into MOF-74 is provided by a distinct absorption peak at 1,020 cm^−1^ (Fig. 1), corresponding to the *ν*(C–N) mode of the amine–metal complex^30^. This peak increases very slowly during ∼10 min as EDA adsorbs on the sample.

The stretch mode of initially adsorbed CO gas (2,170 cm^−1^) does not decrease or shift during and after EDA loading, as would typically occur during co-adsorption of gases inside the MOF (Supplementary Note 2 and Supplementary Fig. 3 for the case of NH_3_ co-adsorption), indicating that the CO molecules not only remain trapped, but also do not interact with the newly added EDA molecules. This observation supports our hypothesis that no EDA molecules penetrate inside the MOF. If EDA interacted with CO inside MOF-74, the CO stretch frequency would be shifted either due to displacement to a secondary binding site or to interaction with EDA. To quantify this statement *ab initio* calculations were performed (Supplementary Fig. 4 and Supplementary Table 1) and show that, if EDA penetrated inside the MOF, the CO-binding energy would be changed only by ∼3 kJ mol^−1^ and its frequency would be shifted by 5–9 cm^−1^, which is not observed.

The above observations and analyses lead us to conclude that pre-adsorbed CO and post-loaded EDA molecules are spatially separated, with EDA residing on the periphery of the MOF microcrystals (after replacing CO molecules only in the outermost pores, since the EDA *E*_binding_=125 kJ mol^−1^>>CO *E*_binding_=52.7 kJ mol^−1^) and acting as a cap that confines pre-loaded CO molecules inside the MOF. However, a direct experimental confirmation of EDA localization is needed.

**Figure 2:**
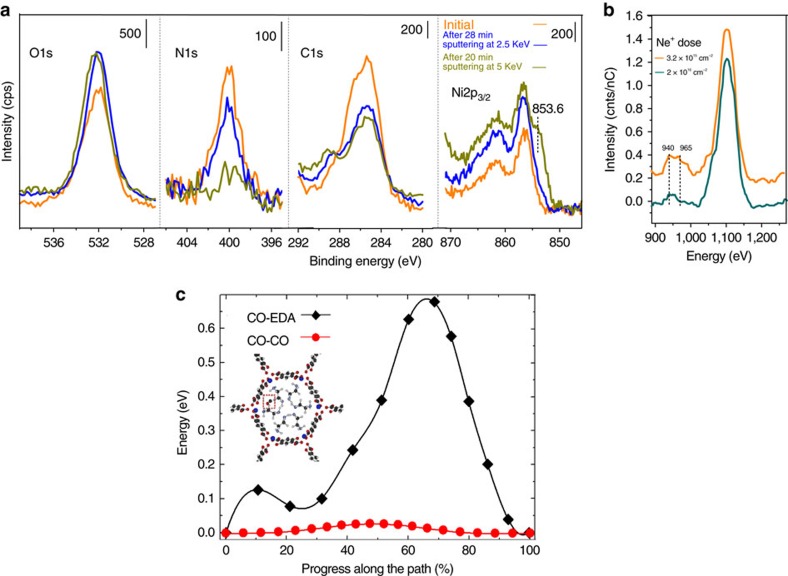
XPS and LEIS characterization of EDA monolayer layer and diffusion profiles of CO molecules. (**a**) XPS spectra of Ni-MOF-74 with post-loaded EDA, before (orange curve) and after being sputtered at 2.5 keV for 28 min (blue curve) and 5 keV for 20 min (dark green curve). (**b**) LEIS spectra of Ni-MOF-74 with post-loaded EDA, after gentile sputtering with a dose of 3.2 × 10^15^ and 2.2 × 10^16^ cm^−2^ Ne^+^ ions. A slight red shift of the peaks after the second sputtering (10 eV corrected in the Figure), most likely resulting from sample charging due to the ion exposure and insulating nature of the MOFs, has been corrected. (**c**) Energy barrier for the diffusion of a CO molecule along the 1D channel of Ni-MOF-74. Red circles: all the metal centers are saturated with CO (Supplementary Fig. 7). Black diamonds: all the metal centers are saturated with EDA molecules. The inset in **c** shows the relaxed atomic position of a CO molecule at the middle of the Ni-MOF-74 channel, where all the adsorption metal sites have been saturated with EDA molecules. The dashed red box shows the CO molecule. Black, red, white, grey and blue spheres represent C, O, H, N and Ni atoms, respectively.

To test and quantify whether EDA is localized at the surface (that is, only the outmost pores) of the MOF crystallites, we have combined X-ray photoelectron spectroscopy (XPS), a surface sensitive technique, with argon GCIS that provides gentle removal of surface atoms (particularly appropriate for organic materials, see ‘Methods' section). Specifically, clusters of ∼2,500 Ar atoms can be generated and charged, then accelerated onto the surface (for example, with 2.5–5 keV). Upon reaching the surface, the cluster decomposes, dividing its kinetic energy among all the Ar atoms, that is, each atom carries ∼1–2 eV kinetic energy. Consequently, these atoms can only remove surface atoms and do not disturb underlying bulk atoms of the rather fragile MOF structure^31^. After each sputtering cycle, XPS data (Ni2*p*_3/2_, C1*s*, N1*s* and O1*s* peaks) are recorded on the sample post-loaded with EDA right after the gas exposure measurement (orange line in Fig. 2a) and after sputtering at 2.5 keV for 28 min (blue line in Fig. 2a), and 5 keV for 20 min (brown line in Fig. 2a). Since the MOF contains only O, Ni and C atoms, a comparison of the N1*s* core level (N is only contained in EDA) with O1*s*, Ni2*p*_3/2_, and C1*s*, provides information on the depth distribution of EDA. While sputtering at 2.5 keV (∼1 eV per Ar atom) for 28 min only partially removes EDA (N1*s* signal), sputtering with 5 keV (∼ 2 eV per Ar atom) for 20 min fully removes nitrogen. The remaining minor feature in the N1*s* spectral region is due to plasmon oscillations of K2*s* of the KBr substrate (Supplementary Fig. 5). The oxygen and Ni signals remain essentially unchanged. The initial decrease of the C signal is associated with the removal of adventitious hydrocarbons physisorbed on the MOF surface. Note that the intensities of N, O and Ni increase slightly after the initial sputtering as screening by adventitious carbon is removed. Thereafter, the C1*s*, O1*s* and Ni2*p*_3/2_ signals remain constant. The shoulder at 853.6 eV in the Ni2*p*_3/2_ peak after removal of EDA is tentatively attributed to surface reconstruction of the Ni corner atoms due to displacement (perturbation) of surface atoms. Importantly, all the above observations clearly point to the localization of EDA at the periphery (surface region) of the MOF microcrystals.

To further verify the localization of EDA at the periphery of the microcrystals, we performed low-energy ion scattering (LEIS) measurements of EDA-pretreated MOF powders. The ultra-shallow penetration depth of this technique (∼1 nm) makes it particularly sensitive to elements at the surface. The spectra are recorded with 3 keV He^+^ ions, and sputtering is performed with 5 keV Ne^+^ ions. Figure 2b shows that, after removing adventitious carbon with a dose of 3.2 × 10^15^ cm^−2^ Ne^+^ ions, there is a clear peak associated with N at ∼950 eV in addition to the O peak at 1,100 eV (Supplementary Fig. 6). The N peak has two components: a surface peak at 960 eV and a subsurface peak at 940 eV, the latter being attributed to EDA at grain boundaries or on tilted surfaces. The surface peak completely disappears after a dose of 2.2 × 10^16^ cm^−2^ Ne^+^ ions, confirming that it is located only at the surface well within 1 nm. Additional sputtering does not appreciably change the relative intensity of the N signature, confirming that it originates from EDA at grain boundaries or tilted surfaces. Together, the XPS and LEIS measurements indicate that EDA forms a monolayer (<1 nm thick) at the surface of the MOF microcrystals.

This knowledge makes it possible to model the EDA arrangement within the Ni-MOF-74 unit cell using *ab initio* calculations. We find that the structure shown in Supplementary Fig. 7a is the most stable and that the binding energy per EDA molecule increases from 125 kJ mol^−1^ for ∼0.17 EDA per Ni^2+^ (1 EDA per unit cell) to 141 kJ mol^−1^ for 1 EDA per Ni^2+^ (1 EDA per metal centre, that is, saturation). This stabilization of aggregated EDA molecules arises from H bonding of the head amine groups (that is, those pointing to the centre of the unit cell, not strongly bonded to the metal centres), as detailed in Supplementary Fig. 7a. These findings are consistent with previous *ab initio* calculations performed in Mg-MOF-74 in which the binding energy was found to increase monotonically with loading from 95 kJ mol^−1^ at ∼0.17 EDA per Mg^2+^ to 125 kJ mol^−1^ 1 EDA per Mg^2+^ (ref. 12). There is thus a significant energy benefit to form a complete layer due to EDA clustering and we conclude that a full EDA layer is completed within the first unit cell of the MOF. Once the top surface (<1 nm) is sealed with a complete layer, further EDA diffusion is not possible due to severe steric constraints. Note that other alkyl amine molecules (for example, trimethylenediamine, n-propylamine, ethanolamine) are less effective than EDA molecules to retain small molecule CO (Supplementary Note 3 and Supplementary Fig. 8). This further indicates that the head NH_2_ groups are crucial to aggregate EDA into a complete layer. We further model the diffusion of the CO molecules through the longitudinal channels of Ni-MOF-74, as described in Supplementary Note 4 (Supplementary Fig. 9 and Supplementary Movies 1 and 2). The results (Fig. 2c) show that the CO diffusion barrier increases from 0.028 eV for a CO-loaded MOF to 0.68 eV for MOF with a monolayer of EDA, that is, a 24-times increase, which is consistent with our experimental observations.

While CO is clearly trapped at room temperature, the removal of CO can be completed by mild annealing up to 100 °C under vacuum (pressure<20 mTorr) and EDA remains mostly unperturbed (Fig. 1). The effect of EDA on CO re-adsorption can now be examined (Supplementary Note 5), using the same loading conditions (∼40 Torr). Supplementary Figure 10 shows that the CO uptake is dramatically reduced compared with the pristine activated MOF-74 (EDA-free), taking over 45 min to reach only ∼25% of the CO loading obtained in pristine MOF-74 loaded in ∼30 min.

### Extension to other small molecules

**Figure 3:**
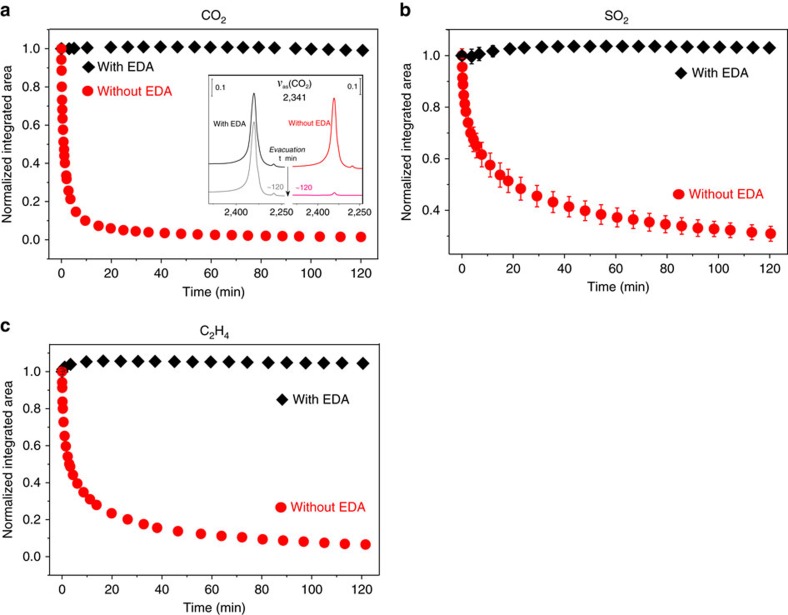
Time evolution of the vibrational bands *ν*_as_(CO_2_) *ν*_as_(SO_2_) and *δ*(CH_2_) upon evacuation. (**a**) Normalized integrated area of CO_2_ asymmetric stretching mode *ν*_as_ upon evacuation (<20 mTorr) in pristine (red circles) and EDA post-loaded (black diamonds). The spectral evolution of *ν*_as_(CO_2_) bands is shown in Supplementary Fig. 11. The error bar of normalized integrated area does not exceed ∼0.025 for the intensity determination of the *ν*_as_(CO_2_) band. The inset of **a** shows the spectra of *ν*_as_(CO_2_) band at *t*=0 and ∼120 min. (**b**) Normalized integrated areas of SO_2_ asymmetric stretching mode *ν*_as_ upon evacuation (<20 mTorr) in pristine (red circles) and EDA post-loaded (black diamonds), The spectral evolution of the *ν*_as_(SO_2_) is shown in Supplementary Fig. 12. The larger error bar in **b** for the *ν*_as_(SO_2_) band was due to interferences of the MOF phonon bands, leading to uncertainties in determining the baseline in the difference spectra (Supplementary Fig. 12c). (**c**) Normalized integrated areas of C_2_H_4_ wagering mode *δ* upon evacuation (<20 mTorr) in pristine (red circles) and EDA post-loaded (black diamonds). The spectral evolution of *δ*(CH_2_) bands is shown in Supplementary Fig. 13. The error bar of normalized integrated area does not exceed ∼0.025 for the intensity determination of the *δ*(CH_2_) bands. For the pristine sample, the initial point (that is, *t*=0) is chosen as the peak intensity after evacuation of gas phase (CO_2_, SO_2_, C_2_H_4_) for ∼10 s; for MOFs post-loaded with EDA, the starting point is still after gas removal at the end of EDA exposure (that is, *t*=0); the integrated areas are normalized to the maximum value obtained at *t*=0.

To test whether EDA acts as a cap in general, we have used this method with other small molecules (CO_2_, SO_2_ and C_2_H_4_, see Supplementary Note 6 and Supplementary Figs 11–14) that are also weakly bonded in MOF-74 and rapidly diffuse out at room temperature. As shown in Fig. 3 for Ni-MOF-74 and Supplementary Fig. 15 for Zn, Co-MOF-74, we find that EDA again provides an effective barrier to retain those molecules. Furthermore, the same method was successfully applied to other MOFs structure such as HKUST-1 (ref. 32; Supplementary Figs 16 and 17) to trap CO_2_ and NO, the latter being an active biological molecule^6^.

### Exposure to water vapour and release of adsorbed molecules

**Figure 4:**
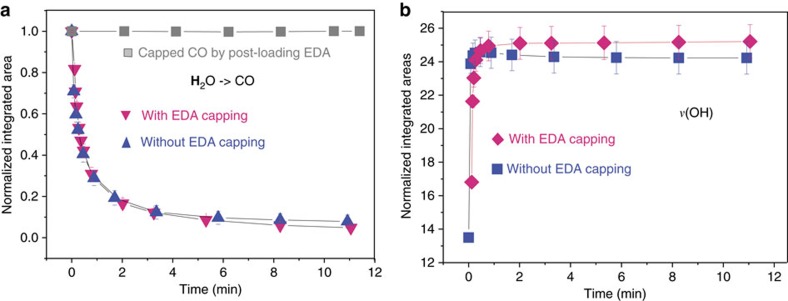
Time evolution of the intensities of the *ν*(CO) and *ν*(OH) bands. (**a**) *ν*(CO) band decay after exposure to 8 Torr H_2_O vapour in pristine (blue triangle) and EDA-capped (pink triangle) Ni-MOF-74. The grey square curve represents the *ν*(CO) band evolution in EDA-capped Ni-MOF-74 under vacuum (<20 mTorr) The error bar of the sharp *ν*(CO) band does not exceed 0.04. (**b**) Water *ν*(OH) band increase in pristine (blue square) and EDA-capped (pink diamond) Ni-MOF-74 at ∼8 Torr vapour phase. The error bar of the *ν*(H_2_O) broad band is larger due to uncertainties in determining the baseline in the difference spectra (Supplementary Fig. 18).

The most striking result was obtained with water molecules, chosen because they can form hydrogen bonds with amine groups: water was observed to pass through the EDA layer without any hindrance and was able to remove pre-adsorbed CO completely. The experiment was started by capping CO molecules in MOFs under 40 Torr by growing an EDA layer via vapour-phase deposition as shown in Fig. 1. After evacuation for ∼1.5 h (that is, CO still retained), 8 Torr vapour-phase H_2_O was introduced into the cell and infrared spectra recorded as a function of time. Figure 4a (and Supplementary Fig. 18) clearly shows that the adsorbed CO peak dramatically weakens while the water stretching band *ν*(OH) quickly strengthens. Clearly, water molecules diffuse into the MOF channel and force the pre-adsorbed CO molecules out through the EDA layer, still present as evidenced by its characteristics *ν*(C–N) band at 1,020 cm^−1^. To quantify the rate of water penetration, the same experiment was performed without EDA capping. CO molecules were loaded into Ni-MOF-74 at 40 Torr for ∼30 min. Followed by a quick evacuation (<3 s), 8 Torr H_2_O was introduced into the cell (Supplementary Fig. 18). Figure 4b shows that there is no measurable difference in the intensity decrease of *ν*(CO) and increase of *ν*(H_2_O) between pristine and EDA-capped MOF, as though the EDA layer did not exist. The dramatically different behaviour of water compared with other gases is tentatively attributed to the ability of water to interact with –NH_2_ through H bonding.

**Figure 5:**
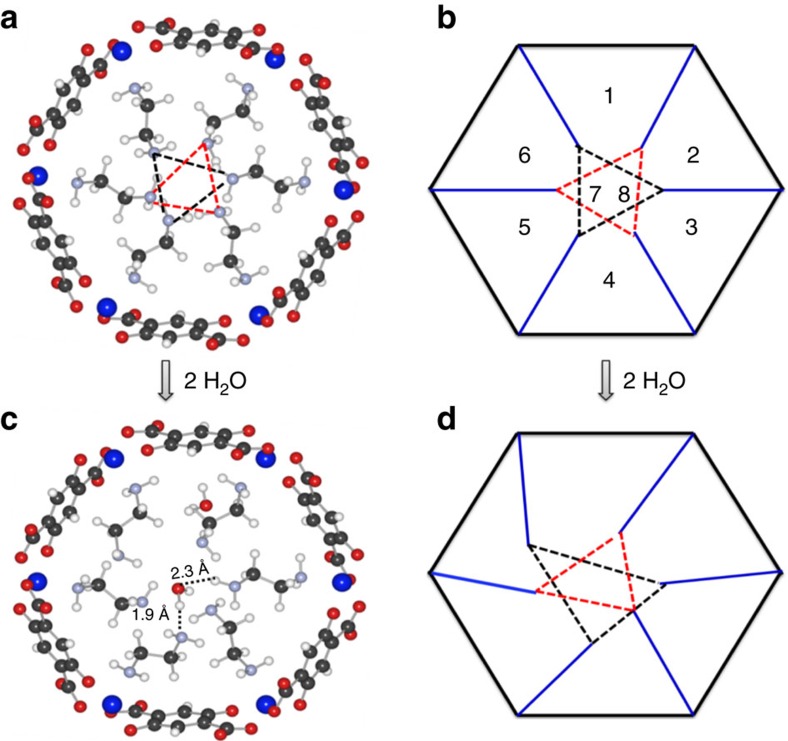
Relaxed atomic position of EDA molecules inside Ni-MOF-74 channel. Black, red, white, grey and blue spheres represent C, O, H, N and Ni atoms, respectively. (**a**) Configuration of 6 EDA molecules before loading 2 H_2_O molecules. (**b**) Structural scheme of the Ni-MOF-74 loaded with the 6 EDA molecules, which are represented as blue lines. The numbers in the figure represent eight possible adsorption sites for the H_2_O molecules. (**c**) Configuration of EDA molecules after adding 2 H_2_O molecules into site 1 and 8. (**d**) Perturbation of triangle areas (empty space) induced by loading 2 H_2_O molecules into site 1 and 7.

To examine this hypothesis, we have investigated the perturbation of the EDA adsorption geometry upon adding water molecules by *ab initio* calculations. When MOF channels are fully loaded with EDA molecules, the –NH_2_ head groups of the adsorbed EDA molecules point towards the centre of the MOF represented as blue lines in Fig. 5b. There are six –NH_2_ divided into two sets, each one of them making an imaginary triangle with N atoms at the apex in the middle of the channel, see the black and red triangles. These two triangles are located in planes parallel to the page, but not in the same plane. There are six adsorption sites for water near the linkers (1–6), and two in the middle of the channel (7 and 8). We observe in Fig. 5c,d that the addition of H_2_O molecules clearly enlarges the triangles. For instance, two water molecules placed at sites 1 and 7 increase the area of the triangle by tilting the –C–C– and –C–N– bond angles of several EDA molecules away from the centre of the channel (Fig. 5d and Supplementary Fig. 19). These water adsorption states are energetically favourable (Supplementary Fig. 20) since water molecules establish the hydrogen bonding with –NH_2_ group, evidenced by the short H…N or H…O distance (Fig. 5c). By continually adding water molecules up to 4 and 6, the area of these triangles in most cases becomes significantly larger (Supplementary Note 7 and Supplementary Figs 19 and 21), enabling water molecules more easily to enter through the channel. This ‘gate opening mechanism' also works for other MOF structures, leading for instance to the removal of NO molecules from within HKUST-1 by water exposure. (Supplementary Fig. 22).

## Discussion

We have demonstrated, combining *in situ* Fourier transform infrared (FTIR) and *ab initio* simulations, that small molecules such as CO, CO_2_, SO_2_ and C_2_H_4_ can be efficiently trapped inside the pores of the MOF-74 system simply by introducing EDA vapour at the end of the loading process. This method avoids the need to perform complex modification of the MOF to increase the internal binding energies for each gas molecule. The EDA molecules are able to block the release of several small molecules from the 1D channels of MOF-74 due to their propensity to agglomerate and organize themselves into a hydrogen-bonded network within the outermost unit cell. This generic approach is also applicable to other MOFs structures and overcomes the limitation of weak interactions between guest molecules and the porous materials. We have further shown that water molecules can easily penetrate this molecular EDA barrier layer and displace previously trapped gas, thus providing a novel method to release trapped gases at room temperature. The understanding of this selective EDA membrane, derived from combined *in situ* measurements and *ab initio* calculations, opens up new avenues for gas storage, delivery and separation, and suggests new applications for molecular membranes.

## Methods

### Synthesis of MOFs samples

The MOFs samples including Ni-MOF-74 Co-MOF-74, Zn-MOF-74 and HKUST-1 are synthesized by following the modified procedure (Supplementary Methods) from refs 32, 33. The crystal structures are confirmed by comparing XRD pattern (Supplementary Fig. 23) and Raman spectra (Supplementary Fig. 24 and Supplementary Note 8) with literature report. After thorough solvent exchange as described in Supplementary Methods, the surface areas reach 913, 1,077, 774 m^2^ g^−1^ for Ni-MOF-74, Co-MOF-74, Zn-MOF-74 (ref. 34), respectively, consistent with the values reported in the original literature^33^.

### *In situ* infrared spectroscopy

All infrared spectroscopic data presented are taken by using a Nicolet 6700 FTIR spectrometer (purchased from Thermo Scientific Inc., USA) equipped with a liquid N_2_ -cooled mercury cadmium telluride MCT-A detector. A high-pressure cell, purchased from Specac Ltd., UK (product number P/N 5850c), is placed in the sample compartment of the infrared spectrometer with the sample at the focal point of the beam. The MOFs (powder, ∼2 to ∼5 mg) are gently pressed onto a KBr pellet (∼1 cm diameter, 1–2 mm thick) and placed in the high-pressure cell. The cell is connected to different gas lines (EDA vapour, NH_3_, CO, CO_2_, SO_2_, CH_2_CH_2_ and so on) for exposure and a vacuum line for evacuation. A pre-chamber is installed close to the cell to mix EDA vapour with other gases (see the diagram in Supplementary Fig. 25). The samples are then activated by evacuation (base pressure<20 mTorr) at 180 °C for at least 3 h and then cooled back to room temperature for gas exposure measurements. All spectra are recorded in transmission mode from 650 cm^−1^ (MCT-A) to 4,000 cm^−1^ (4 cm^−1^ spectral resolution).

### X-ray photoelectron spectroscopy and gas cluster sputtering

X-ray photoelectron measurements were performed in conjunction with gas cluster ion beams, initially developed in the late 90's (ref. 35). The principle for sputtering with individual Ar atoms has been well described^36^. GCIS is particularly attractive to gently remove the top layers of fragile organic materials^37^,^38^,^39^,^40^. It has been used in conjunction with XPS to explore the depth distribution of atoms^41^. When standard Ar+ sputtering is used (∼1 keV per Ar+ ion), there is considerable perturbation of the MOF with substantial preferential removal of O and C relative to Ni (not shown), which makes it impossible to determine the location of EDA. Therefore, Ar GCIS is used in removing the surface EDA molecules on MOFs sample. A large cluster (∼2,500 Ar atoms) is generated and charged by removal of 1 electron, then accelerated by a 2.5 or 5 keV potential difference. Upon impact, the kinetic energy of the cluster is distributed among all Ar atoms (that is, ∼1 or 2 eV per atom), which is insufficient to penetrate into the MOF, limiting the sputtering to surface species only. The incidence angle of the cluster is 45°, the bombarded area is 1 × 1 mm^2^, and the sample is rotated at a rate of 0.2 r.p.m. for 5–15 min and then 0.5 r.p.m. for 2 min to achieve a uniform sputtering. All the data were recorded with charge compensation. For XPS measurements, the MOFs pellet used for infrared measurements is taped on the puck with double sided tape. A Al kα monochromated source is used with a beam size of 200 × 200 μm^2^. Spectra are recorded at a 45° takeoff angle with respect to the surface. The base pressure is typically below 4 × 10^−8^ Pa and the Ar pressure during the sputtering is 2 × 10^−6^ Torr.

### Low-energy ion scattering

LEIS measurements are performed using a Qtac analyzer (IonTOF Gmbh, Münster, Germany) using 3 keV He^+^ and 5 keV Ne^+^ as the probe and sputtering ions, respectively. The He^+^ current used for the measurements is ∼4 nA, and the Ne^+^ sputtering current is ∼11 nA. The instrument employs a double-toroidal analyzer that collects all ions scattered within an angular range of 144–146° and images them according to their energy onto a position sensitive detector. Samples for LEIS are prepared by pressing the EDA-pretreated MOF powders into a tungsten mesh and mounting the mesh onto an SiO_2_/Si wafer. A 1.5 × 1.5 mm^2^ sample area is analyzed. Ne+ sputtering is performed using the LEIS ion gun, and thus the ions impinge at normal incidence on the sample, unlike the conventional 45° sputtering geometry.

### *Ab initio* calculations

*Ab initio* calculations are performed at the density functional theory level, as implemented in Quantum Espresso^42^. To correctly capture the crucial van der Waals interaction between the MOF and the guest molecules, we use the non-local functional vdW-DF (refs 43, 44, 45, 46). Ultra-soft pseudopotentials are used with cutoffs of 544 and 5,440 eV for the wave functions and charge density, respectively. Due to the large dimensions of the unit cell, only the Γ-point is sampled. To model the diffussion process we use a transition-state search algorithm, that is, the climbing-image nudged-elastic band method^47^,^48^. This method is chosen because it finds the lowest-energy pathway between an initial and final state, which may well deviate from a straight line (that is, linear interpolation) between the two. Furthermore, this method allows us to obtain a clear picture of the interaction between the CO molecule and the EDA molecules blocking the pores, which cannot easily be obtained by other methods such as *ab initio* molecular dynamics. We start from the experimental rhombohedral structure of Ni-MOF-74 with 54 atoms in its primitive cell and space group 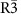. The description through hexagonal axes is *a*=*b*=25.719 Å and *c*=6.741 Å (ref. 49), and *α*=*β*=90° and ***γ*****=**120°. We optimized all atomic positions until the forces are <2.6 × 10^−4^ eV Å^−1^.

### Data availability

The data that support the findings of this study are available from the corresponding author upon request.

## Additional information

**How to cite this article:** Tan, K. *et al*. Trapping gases in metal-organic frameworks with a selective surface molecular barrier layer. *Nat. Commun.* **7,** 13871 doi: 10.1038/ncomms13871 (2016).

**Publisher's note:** Springer Nature remains neutral with regard to jurisdictional claims in published maps and institutional affiliations.

## Supplementary Material

Supplementary Information Supplementary Figures, Supplementary Table, Supplementary Notes, Supplementary Methods and Supplementary References.

Supplementary Movie 1 CO molecule diffusing through Ni-MOF-74 channel where the metal centers are saturated with other CO molecules

Supplementary Movie 2 CO molecule diffusing through Ni-MOF-74 channel where the metal centers are saturated with ethylenediamine (EDA) molecules

Peer Review File
