# Supplementary material for: Trapping gases in metal-organic frameworks with a selective surface molecular barrier layer
Source: Nat Commun. 2016 Dec 13;7:13871. doi: 10.1038/ncomms13871 (PMC5159845; doi:10.1038/ncomms13871)
Supplement: Supplementary Information — Supplementary Figures, Supplementary Table, Supplementary Notes, Supplementary Methods and Supplementary References. [file ncomms13871-s1.pdf]

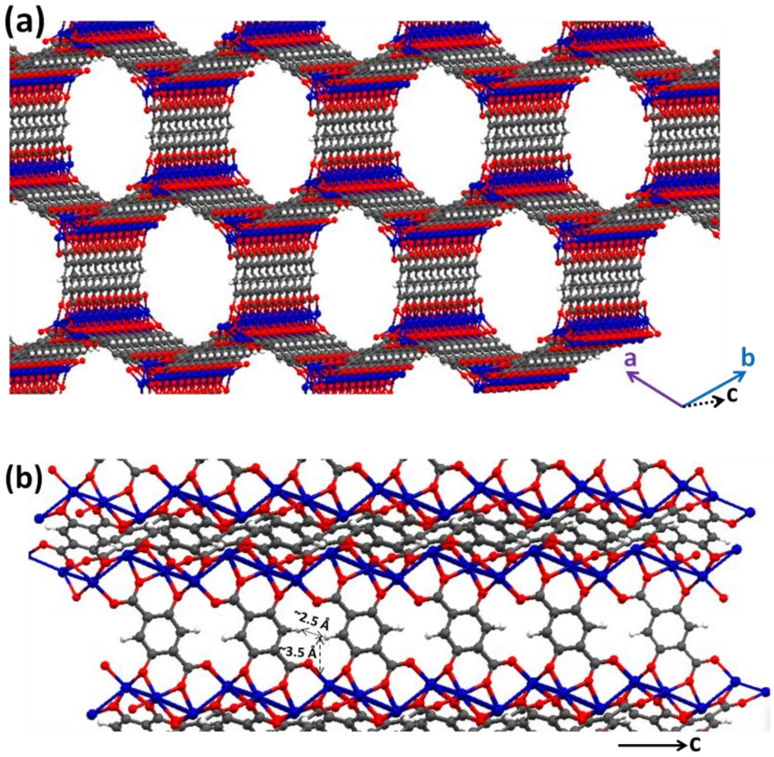

Supplementary Figure 1 / Crystal structure of Ni-MOF-74. (a) side view along c axis of hexagonal cell. (b) view perpendicular to the c axis. Color scheme: blue, grey, red, and white spheres represent Ni, C, O, and H atoms respectively. The interatomic space between two nearest neighbor H atoms belonging to two adjacent linkers is ~2.5 Å.

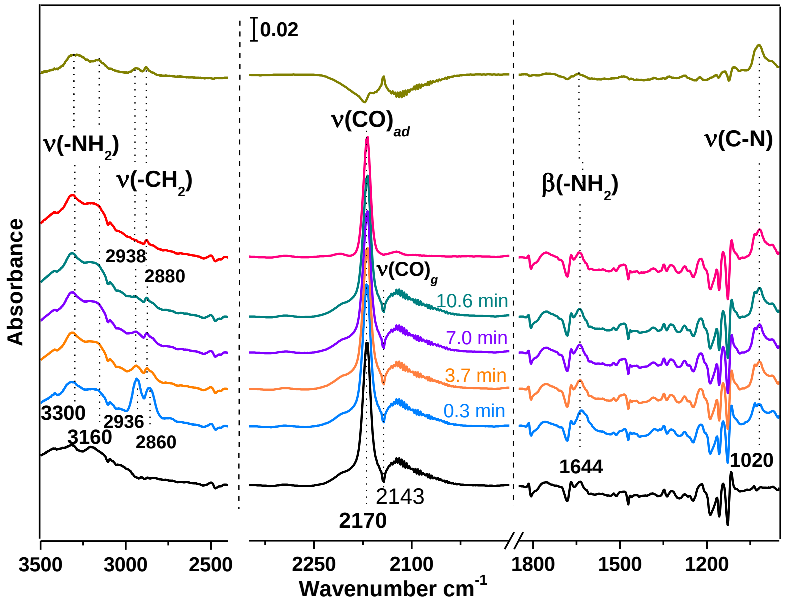

Supplementary Figure 2 /. Evolution of infrared spectra of pre-loaded CO molecules in Ni-MOF-74 after introducing a gas mixture of CO and EDA (~40 Torr CO / ~4 Torr EDA). The bottom black spectrum shows the pure CO adsorption after an equilibrium of ~30 min. The middle five spectra show the time dependence features after introducing mixture of CO + EDA for ~10 min and subsequent evacuation within ~10 sec (pink). All the spectra are referenced to the activated MOFs in vacuum except for the top differential spectrum, which is obtained by subtracting the spectra after evacuating mixture gas phase of CO and EDA (pink) and before loading EDA (black).

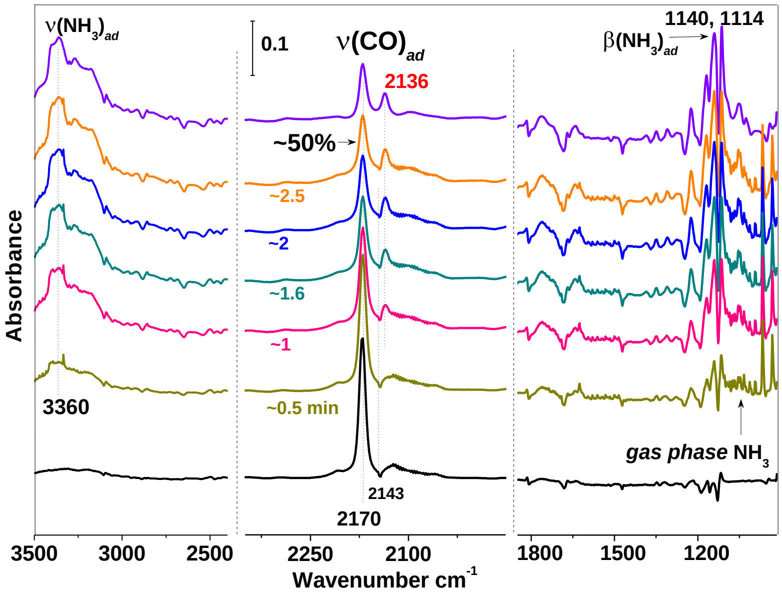

Supplementary Figure 3 / Evolution of infrared spectra of pre-loaded CO molecules in Ni-MOF-74 after introducing mixture gas of CO and NH_3 (~40 Torr CO: ~4 Torr NH_3). The bottom black spectrum shows the pure CO adsorption after an equilibrium of ~30 min. The middle six spectra show the time dependence features after introducing mixture of CO + NH_3 for ~2.5 min and subsequent evacuation within ~10 sec (orange). The adsorbed NH_3 molecules are characterized by the broad ν(NH_3) band around ~3360 cm^{-1} and β(NH_3) around ~1114 to ~1140 cm^{-1}. All the spectra are referenced to the activated MOFs in vacuum.

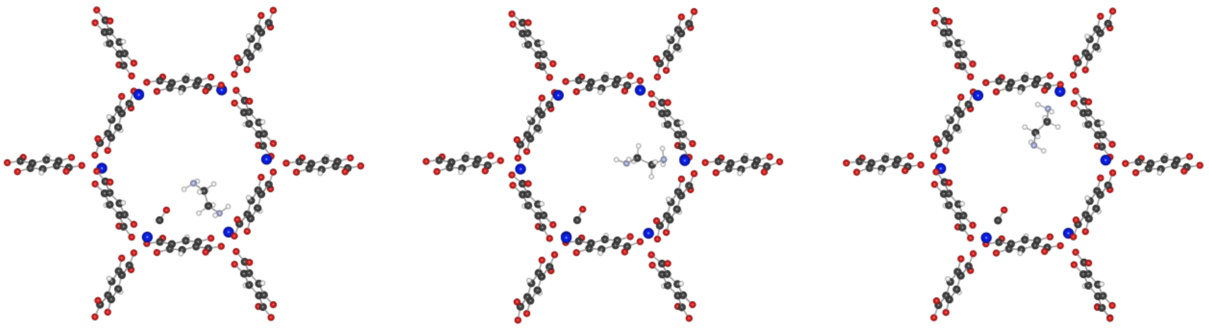

Supplementary Figure 4 / Co-adsorption of CO and EDA at the metal centers of the Ni-MOF-74 system. Left, middle, and right hand side panels show the adsorption of these two molecules as first, second, and third neighbors, respectively. Blue, grey, red, and white spheres represent Ni, C, O, and H atoms, respectively.

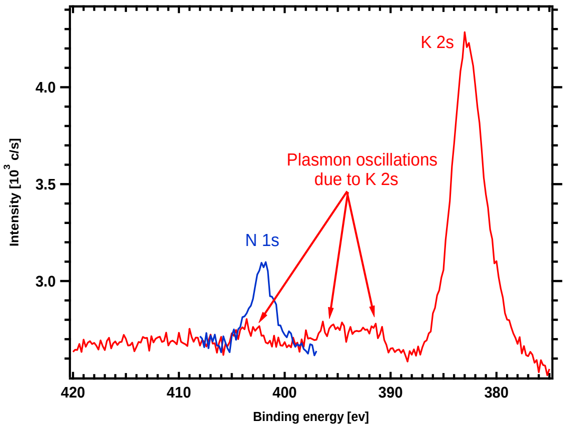

Supplementary Figure 5 / XPS spectra of blank KBr without MOF sample.

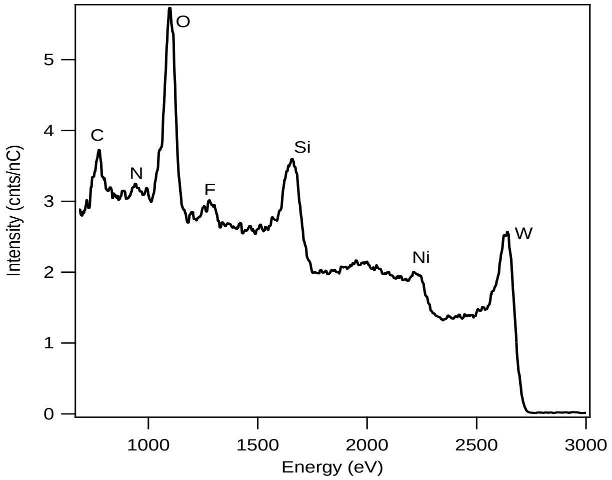

Supplementary Figure 6 / LEIS spectra collected over a broad energy range after successive Ne^+ sputtering with net dose of 2×10^{16} cm^{-2}.

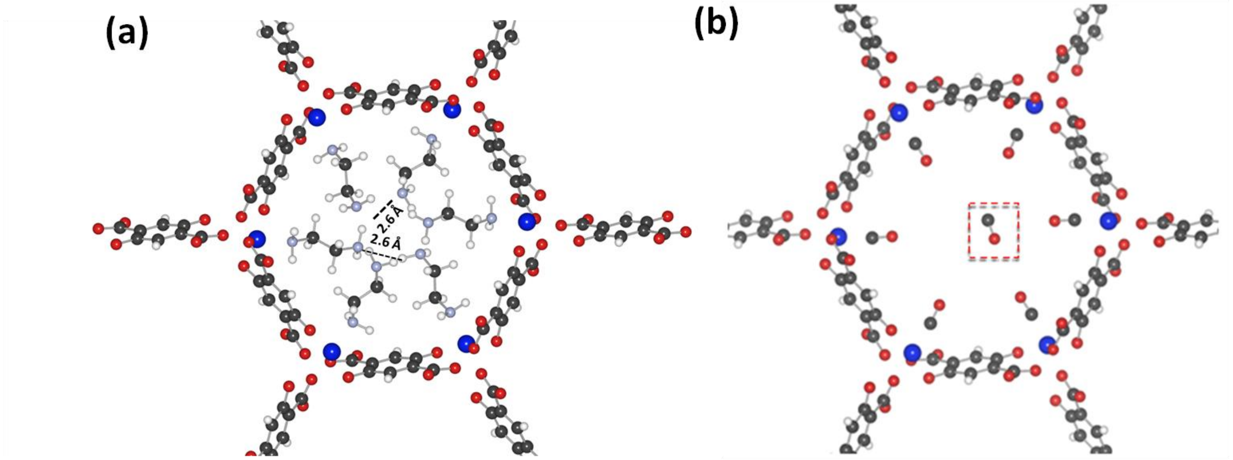

Supplementary Figure 7 / Relaxed atomic position of the Ni-MOF-74 channel where all the adsorption metal cites have been saturated with EDA (a) and CO molecules (b). The dashed red box shows the CO molecule. Black, red, white, grey, and blue spheres represent C, O, H, N, and Ni atoms, respectively.

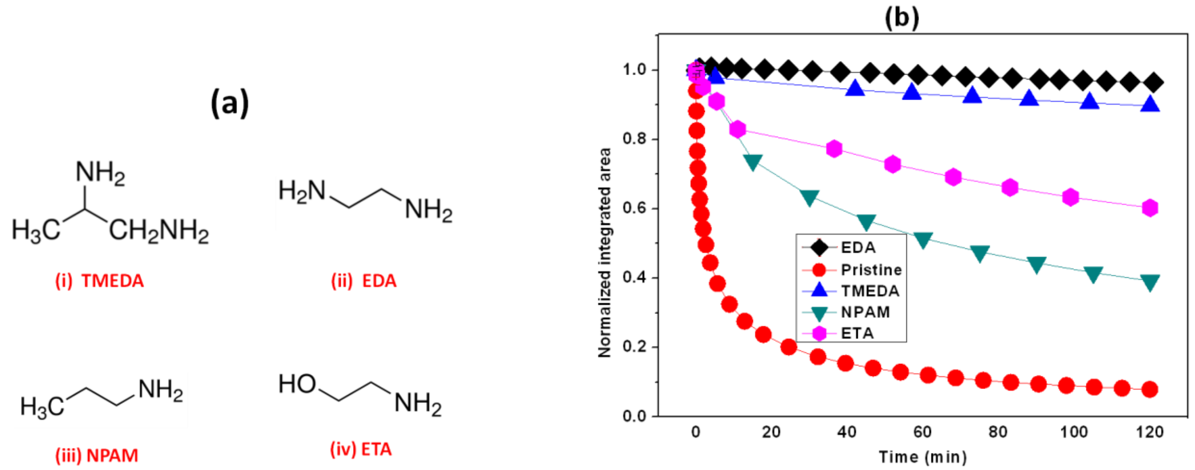

Supplementary Figure 8 / (a) Chemical structure of different alkyl amine molecules: i, trimethylenediamine (TMEDA); ii, ethylenediamine (EDA); iii, n-propylamine (NPAM); iv, ethanolamine (ETA). (b) Time evolution of the ν(CO) band (2170 cm^{-1}) by measuring the integrated areas upon evacuation (< 20 mTorr) for pristine (red circles) and alkyl amine post-loaded samples.

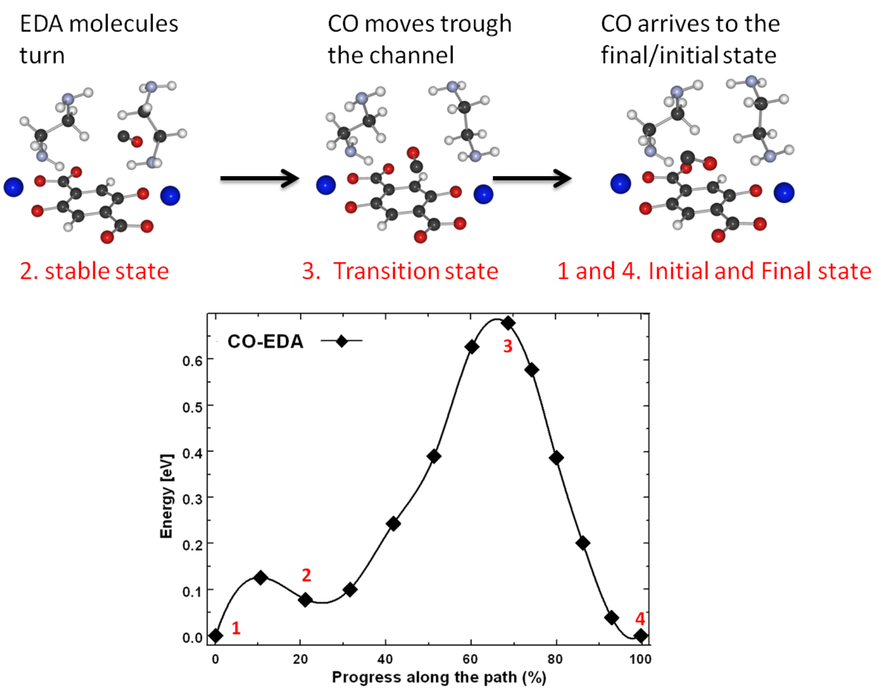

Supplementary Figure 9 / Diffusion progress coordinates of CO molecule through EDA layer. Three figures show the initial state, middle stable state, transition state, and final state.

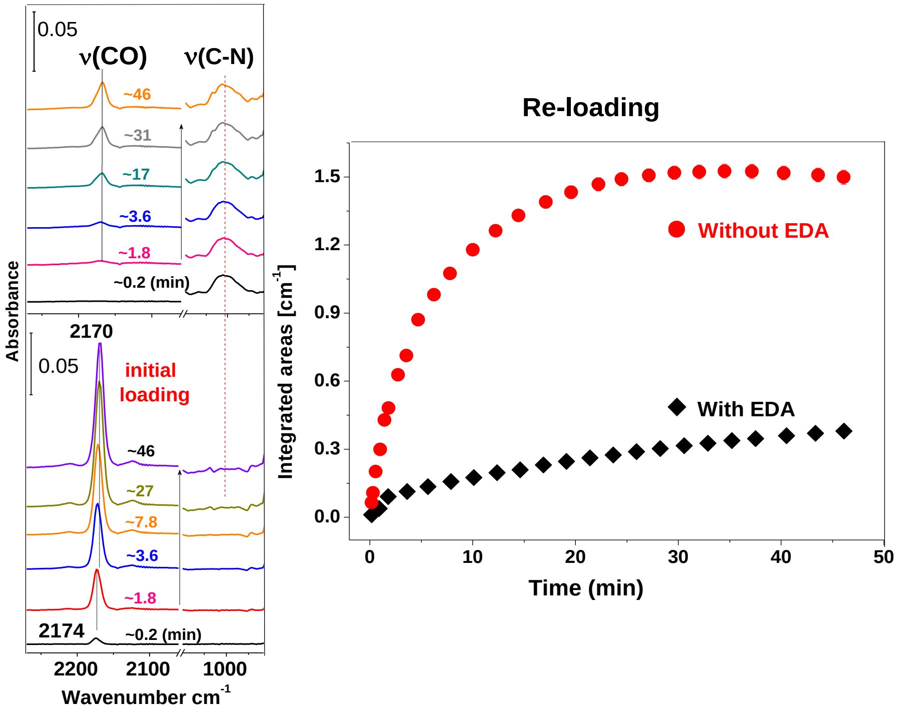

Supplementary Figure 10 / Time evolution of the ν(CO) band upon reloading in ~40 Torr CO in the pristine MOF (without EDA, red circles) and of the same sample after exposure to a CO/EDA mixture and subsequent annealing (Fig.1). All the spectra are collected at 24 °C and referenced to the pristine activated MOF in vacuum. The CO gas-phase contribution is subtracted from each spectrum and the remaining 3% adsorbed CO after annealing (Fig.1) is also subtracted from the spectra collected during the reloading.

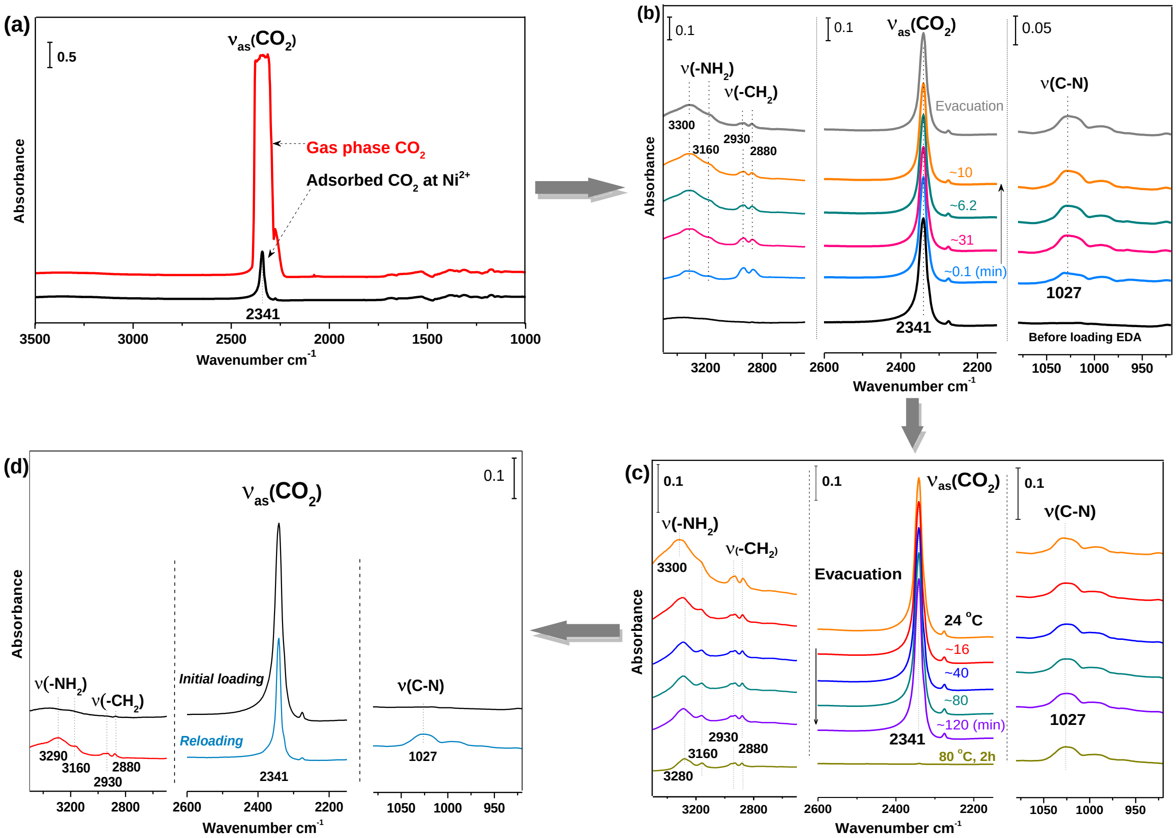

Supplementary Figure 11 / (a) IR spectra of CO_2 adsorption in Ni-MOF-74 at a pressure of ~80 Torr (red) and subsequent evacuation within ~10 sec (black)**.** The broad strong peak is due to the gas-phase CO_2 signal, while the sharp peak at 2341 cm^{-1} is due to the adsorbed CO_2 at open metal site Ni^{2+}. (b) Evolution of infrared spectra of pre-loaded CO_2 molecules in Ni-MOF-74 after being exposed to ~4 Torr EDA. The bottom black spectrum shows the adsorbed CO_2 after subsequent evacuation of ~80 Torr gas phase CO_2 within ~10 sec (panel a). The middle five spectra show the time dependence features after introducing EDA for 10 min. and subsequent evacuation within ~10 sec (grey). All the spectra are referenced to the activated pristine MOFs in vacuum. (c) CO_2 band evolution (2341 cm^{-1}) upon evacuation under vacuum (< 20 mTorr) in Ni-MOF-74 with post-loaded EDA. All the spectra were collected at 24 °C and referenced to the activated MOF in vacuum. The annealing was performed by increasing the temperature to 80 °C, holding for 2 h and cooling back to 24 °C for spectrum collection. (d) IR spectra of reloaded CO_2 in EDA capped Ni-MOF-74 after introducing ~80 Torr CO_2 for ~30 min and subsequent evacuation of gas phase within ~10 seconds, compared to the initial loading in the pristine sample without EDA.

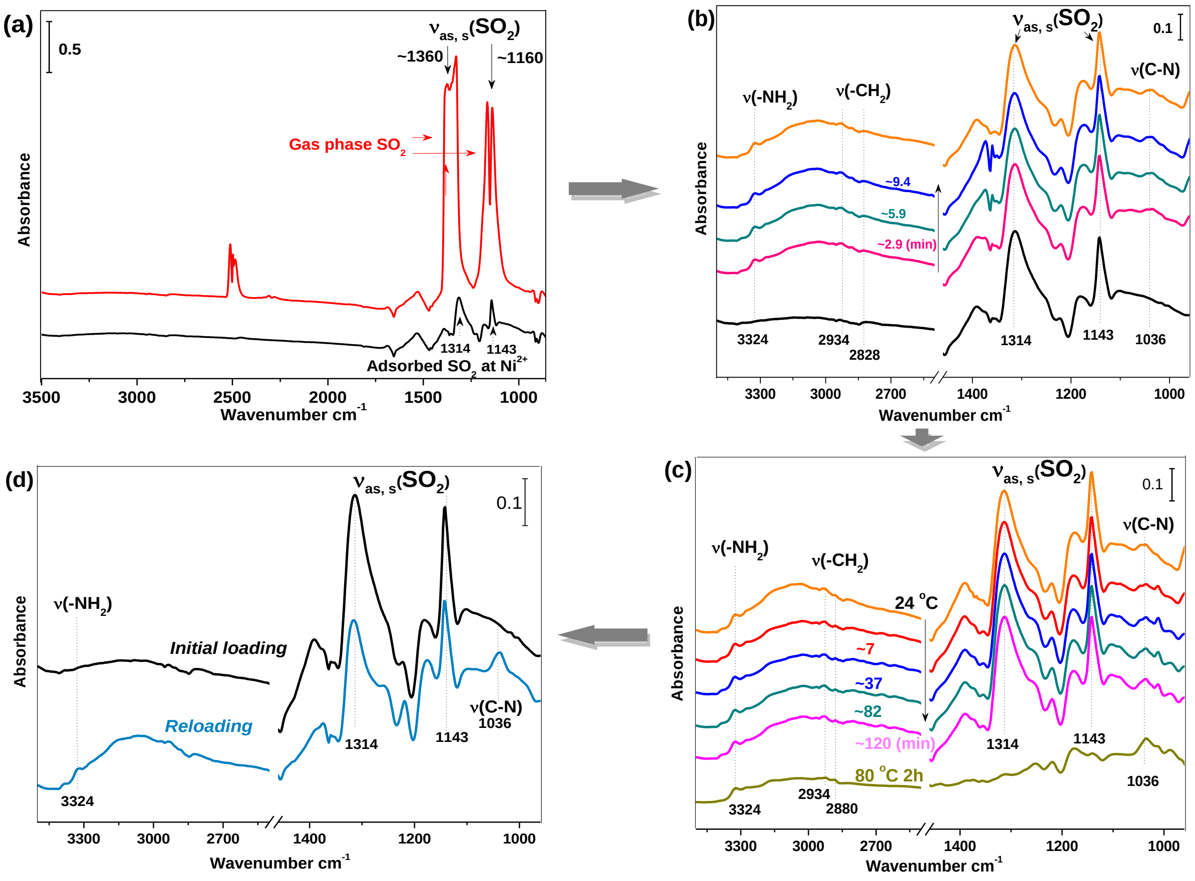

Supplementary Figure 12 / (a) IR spectra of SO_2 adsorption in Ni-MOF-74 at a pressure of ~250 Torr (red) and subsequent evacuation within 10 sec (black). The high intensity bands around ~1360 and ~1150 cm^{-1} are due to the gas phase $\nu_{as}(SO_2)$ and $\nu_s(SO_2)$ spectra. The sharp peaks at 1314 cm^{-1} and 1143 cm^{-1} are due to the asymmetric band (ν_{as}) and symmetric band $\nu_s(SO_2)$ of adsorbed SO_2 at the open metal site Ni^{2+}. (b) Evolution of infrared spectra of pre-loaded SO_2 molecules in Ni-MOF-74 after being exposed to ~4 Torr EDA. The bottom black spectrum shows the adsorbed SO_2 after subsequent evacuation of ~250 Torr gas phase within ~10 sec (Supplementary Fig. 12a). The middle four spectra show the time dependence features after introducing EDA for ~10 min and subsequent evacuation within ~10 sec (orange). All the spectra are referenced to the activated pristine MOFs in vacuum. (c) $\nu_{as}(SO_2)$ band evolution (1314 cm^{-1}) upon evacuation in vacuum (< 20 mTorr) in Ni-MOF-74 with post-loaded EDA. All the spectra were collected at ~24 °C and referenced to the activated MOF in vacuum. The annealing was performed by increasing the temperature to 80 °C, holding for 2 h. and cooling back to ~24 °C for spectrum collection. (d) IR spectra of reloaded SO_2 in EDA capped Ni-MOF-74 after introducing ~250 Torr SO_2 for ~30 min and subsequent evacuation of gas phase within ~10 sec, compared to the initial loading in the pristine sample without EDA.

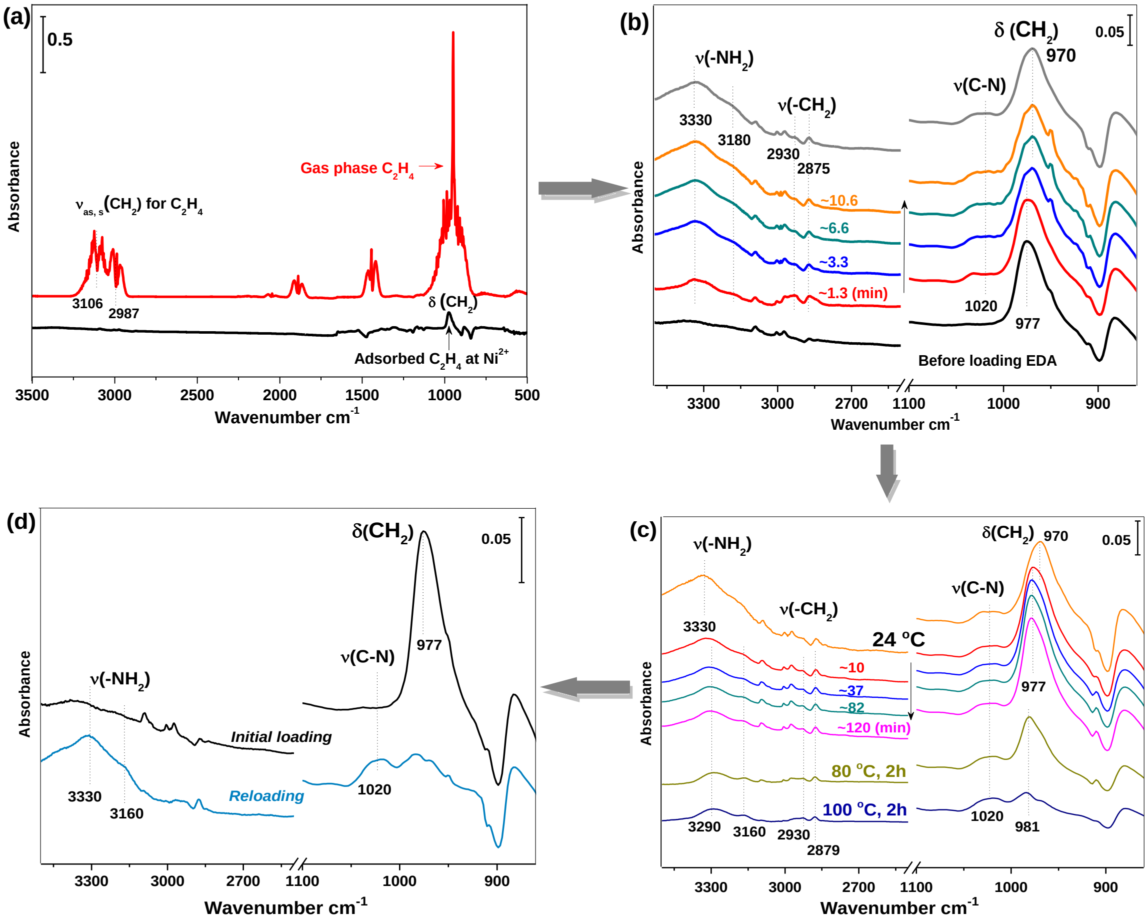

Supplementary Figure 13 / (a) IR spectra of C_2H_4 adsorption in Ni-MOF-74 at a pressure of ~200 Torr (red) and subsequent evacuation within ~10 sec (black). The broad doublet peaks centered at 3106 cm^{-1} and 2987 cm^{-1} in the red spectra are due to the gas phase mode of asymmetric $\nu_{as}(CH_2)$ and symmetric $\nu_s(CH_2)$. The broad peak centered at 949 cm^{-1} is due to the wagging vibration $\delta(CH_2)$ of adsorbed C_2H_4 molecules at open metal site Ni^{2+}. After evacuation of gas phase, the $\nu_{as}(CH_2)$ mode of adsorbed C_2H_4 is weak and overlaps with the derivative feature of ν(–CH) mode of benzene ring of MOF linker. The strong $\sigma(CH_2)$ band falls into the MOF-74's phonon gap[1] and is observable in the black spectrum. (b) Evolution of infrared spectra of pre-loaded C_2H_4 molecules in Ni-MOF-74 after being exposed to ~4 Torr EDA. The bottom black spectrum shows the adsorbed C_2H_4 after subsequent evacuation of ~200 Torr gas phase within ~10 sec (panel a). The middle five spectra show the time dependence features after introducing EDA for ~10 min and subsequent evacuation within ~10 sec (grey). All the spectra are referenced to the activated pristine MOFs in vacuum. (c) $\delta(CH_2)$ band evolution (977 cm^{-1}) upon evacuation in vacuum (< 20 mTorr) for Ni-MOF-74 with post-loaded EDA. All the spectra were collected at 24 °C and referenced to the activated MOF in vacuum. The annealing sequence includes 2 h at 80 °C, cooling back to room temperature for data collection, and an additional 2 h at 100 °C, cooling back to room temperature for spectrum collection. (d) IR spectra of reloaded C_2H_4 in EDA capped Ni-MOF-74 after introducing ~200 Torr C_2H_4 for ~30 min and subsequent evacuation of gas phase within 10 seconds, compared to the initial loading in the pristine sample without EDA.

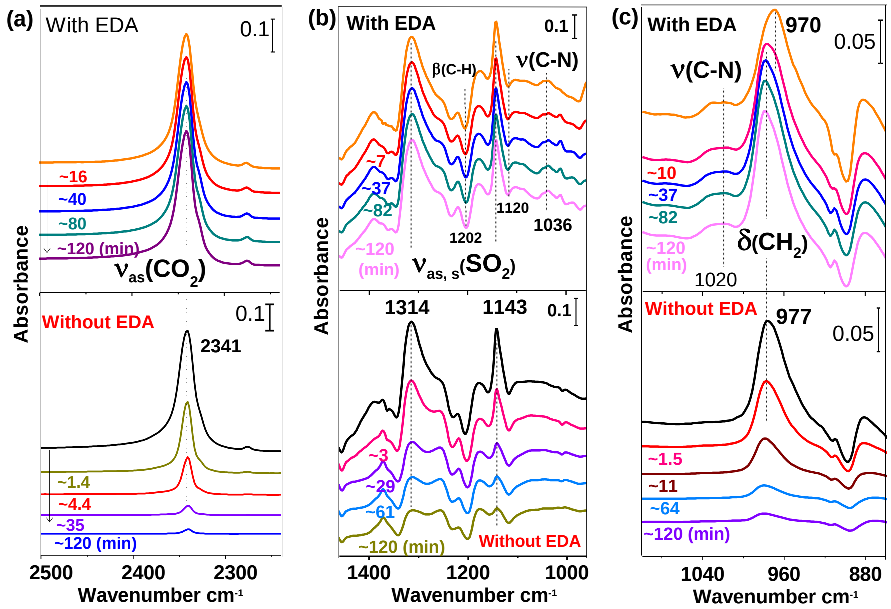

Supplementary Figure 14 / Time dependence spectra of CO_2 (a), SO_2 (b), C_2H_4 (c) desorption under vacuum. The data were collected in Ni-MOF-74 with post-loaded EDA (top panels) and pristine sample without EDA (bottom panels).

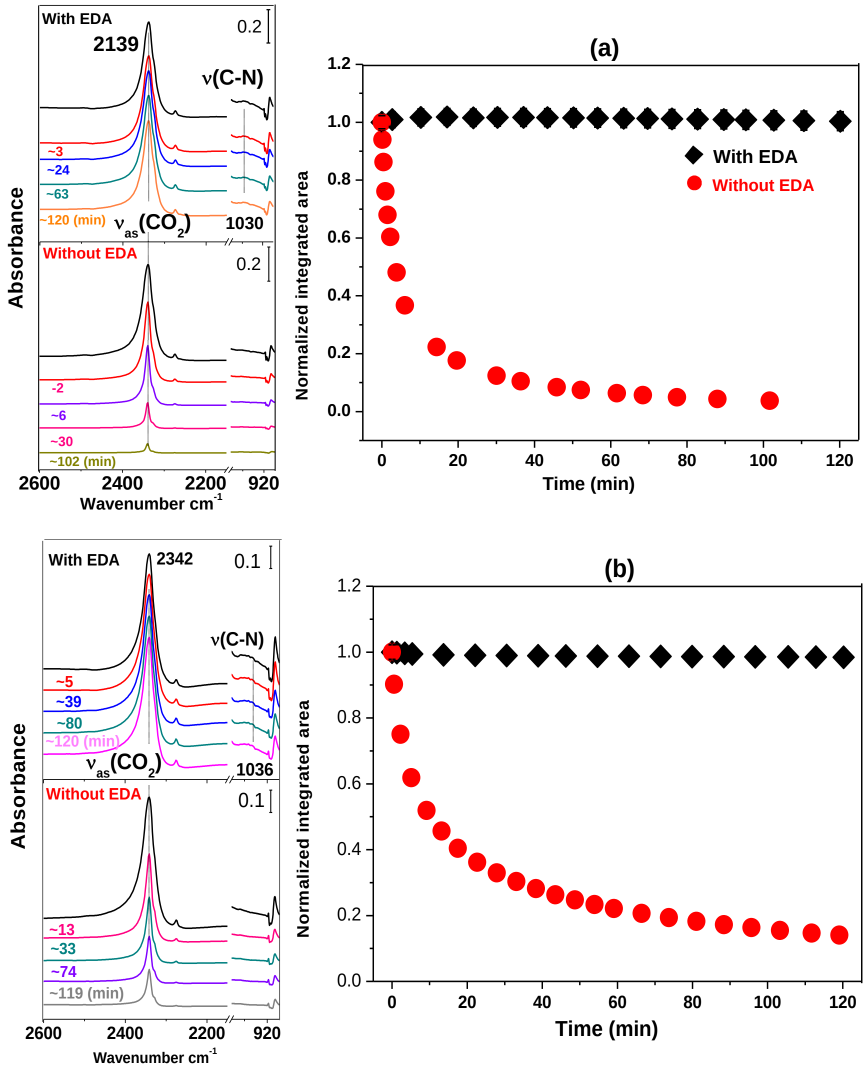

Supplementary Figure 15 / CO_2 band evolution upon evacuation in vacuum (< 20 mtorr) for Co-MOF-74 (panel a) and Zn-MOF-74 (panel b) with post-loaded EDA (black diamonds) and in pristine state (red circles). All the spectra were collected at 24 °C and referenced to the activated MOF in vacuum. The desorption rate was monitored by recording the spectra and the intensity of the $\nu_{as}(CO_2)$ band during desorption. The error bars of normalized integrated area do not exceed 0.03 in both Co, Zn-MOF-74. The procedure for loading CO_2 + EDA in Zn, Co-MOF-74 was the same as for Ni-MOF-74. The concentrations of CO_2 in Zn, Co-MOF-74 under ~80 Torr CO_2 were estimated to be 0.21 and 0.44, respectively.[2]

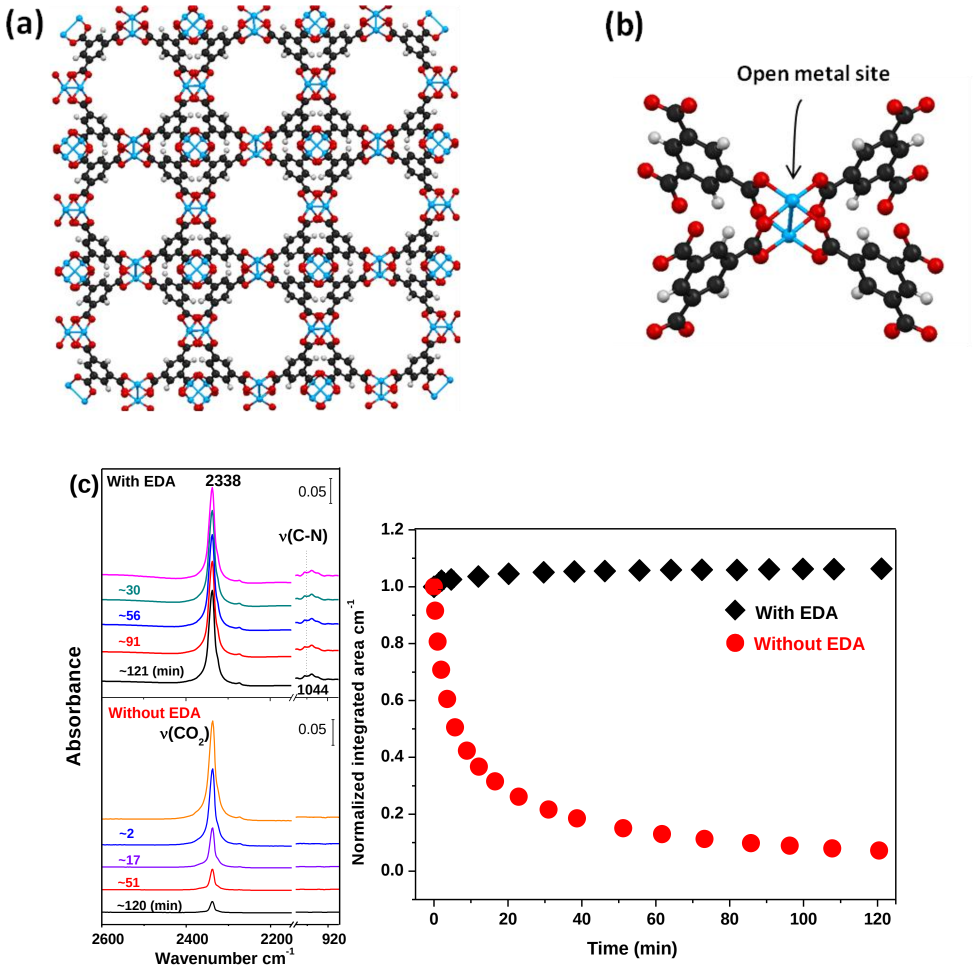

Supplementary Figure 16 / (a) Top view along the [100] direction of the cubic cell of MOF HKUST-1 and (b) the dicopper(II) tetracarboxylate building block . Color scheme: light blue, grey, red, and white spheres represent Cu, C, O, and H atoms respectively. HKUST-1, also called Cu(BTC) (BTC = benzenetricarboxylic acid), has face a centered-cubic crystalline structure and contains an intersecting three-dimensional (3D) system of large square-shaped pores (9 Å x 9 Å). The solvent free structure is composed of paddlewheel dimeric cupric tetracarboxylate units (panel b), in which each copper atom is coordinated by four oxygen atoms, coming from the benzene tricarboxylic acid linkers.[3] The terminal water molecules in the apical sites of the Cu-Cu dimer can be removed by thermal activation, offering coordinative binding vacancies (open metal sites) to the guest molecules such as EDA, CO_2 and NO. The heat of adsorption CO_2 in HKUST-1, derived from temperature dependent isotherms, is 35 kj/mol.[4] (c) ν(CO_2) band evolution upon evacuation in vacuum (< 20 mtorr) for HKUST-1 with (black diamonds) and without (red circles) post-loaded EDA. All the spectra were collected at 24 °C and referenced to the activated MOF in vacuum. The error bars of normalized integrated areas do not exceed 0.03. The loading procedure of CO_2 + EDA (~80 Torr + ~4 Torr) in HKUST-1 is the same as that in MOF-74. The uptake of CO_2 within HKUST-1 sample around ~80 Torr is measured to be 27.3 mg/g.[2] The coordinatively bonded EDA at the Cu^{2+} site is observed at 1044 cm^{-1}, associated with the ν(C-N) mode.

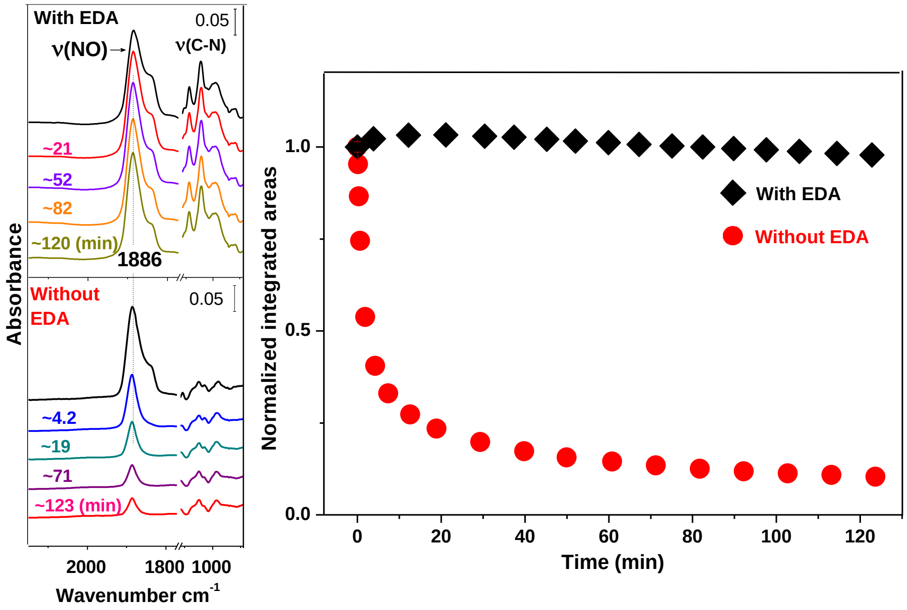

Supplementary Figure 17 / NO band (stretching ν) evolution upon evacuation in vacuum (< 20 mtorr) for HKUST-1 with (black diamonds) and without (red circles) post-loaded EDA. All the spectra were collected at 24 °C and referenced to the activated MOF in vacuum. The desorption rate was monitored by recording the spectra and the intensity of the ν(NO) band during desorption. The error bars of normalized integrated areas for ν(NO) do not exceed 0.02. The loading sequence of NO + EDA (~1000 Torr + ~4 Torr) in HKUST-1 sample is the same as for other molecules in MOF-74 samples. The adsorption uptake of NO in HKUST-1 is over 3 mmol/g at 298 K, determined by isotherm measurements in [5] and by the observed frequency of ν(NO), based on the literature.[5]

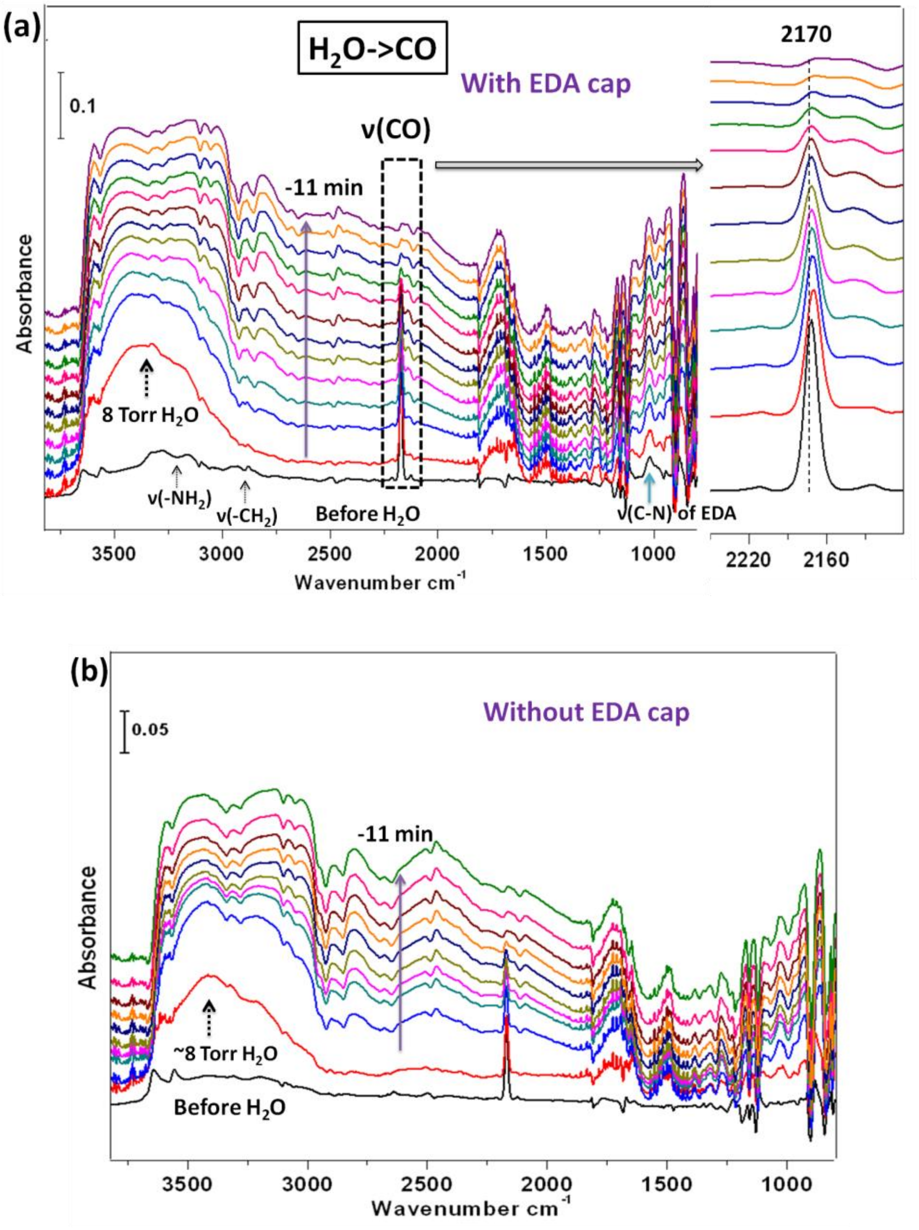

Supplementary Figure 18 / (a) IR absorption spectra of EDA-capped Ni-MOF-74 samples previously loaded with 40 Torr CO before and after exposure to 8 Torr H_2O referenced to pristine (freshly thermally activated in vacuum, < 20 mTorr), as a function of time in 8 Torr water vapor. The broad band above ~2600 cm^{-1} corresponds to adsorbed water and the sharp mode at 2170 cm^{-1} to adsorbed CO. (b) Similar spectra obtained for samples without EDA capping. The bottom spectrum in each case (black) shows that there is a very small amount of water in both samples prior to introducing 8 Torr water vapor.

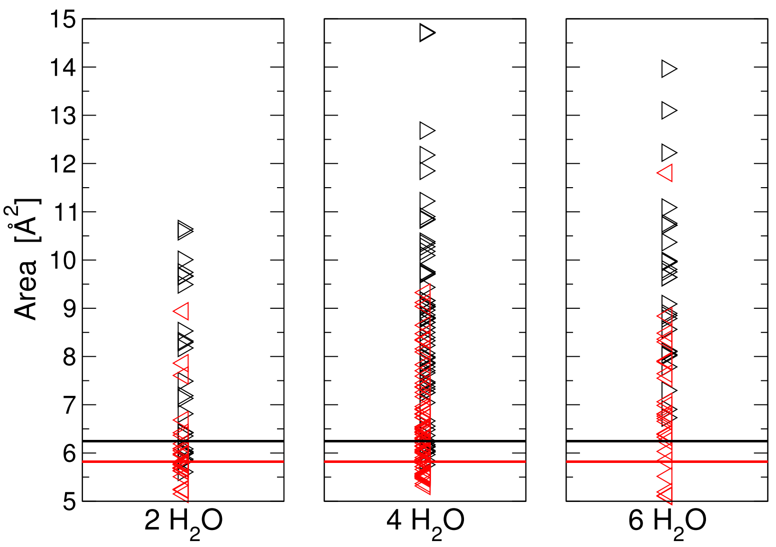

Supplementary Figure 19 / Areas of the black and red triangles depicted in Fig. 5b and 5d upon adding 2, 4, and 6 H_2O molecules. As indicated in Fig. 5b, there are 8 adsorption sites for a water molecule. Thus, there are 28 ways to add 2 H_2O molecules, 70 ways to add 4 H_2O molecules, and 28 ways to add 6 H_2O molecules; results for all those possibilities are given in the three plots. The solid lines represent the areas of the triangles without any water molecules. In the majority of cases a significant increase of the triangular area is observable upon adding water, constituting a gate-opening mechanism that greatly enhances diffusion.

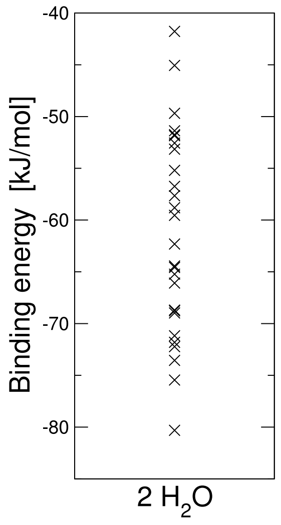

Supplementary Figure 20 / Binding energy of water molecules in EDA-loaded Ni-MOF-74 for the case of adding 2 H_2O molecules. 28 binding energies are reported, corresponding to all possible ways of adding 2 water molecules to the 8 existing adsorption sites, see Supplementary Fig. 21.

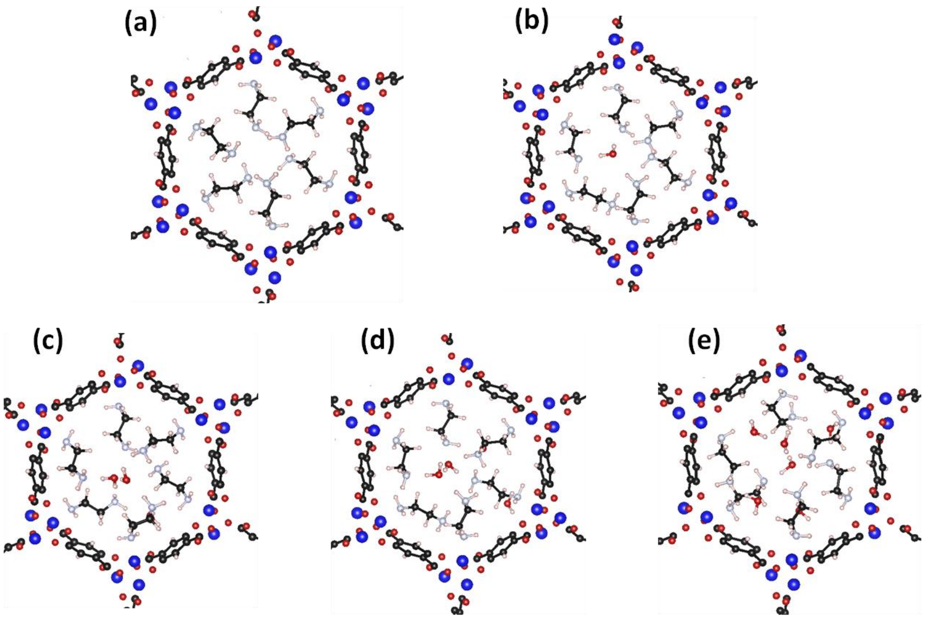

Supplementary Figure 21 / Relaxed atomic position of EDA molecules at the middle of the Ni-MOF-74 channel upon loading H_2O molecule(s). (a), (b), (c), (d), and (e) show addition of 0, 1, 3, 4, and 6 water molecules. Water molecules can be added at many different places and we show here the structures corresponding to the largest perturbation on the system, i.e. structures that show the largest deviation from (a).

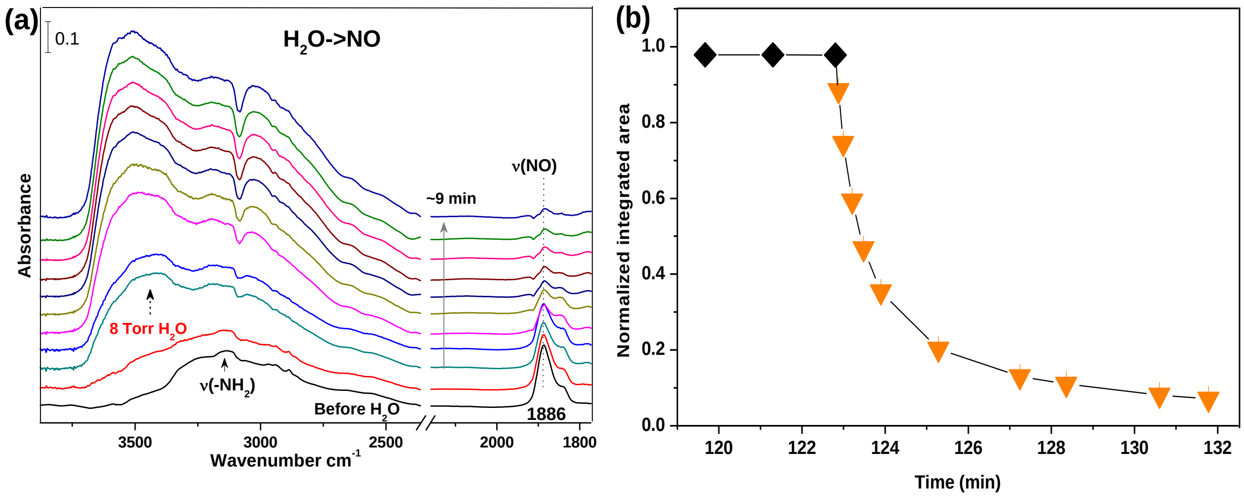

Supplementary Figure 22 / (a) IR absorption spectra of an EDA-capped HKUST-1 sample previously loaded with ~1000 Torr NO before (Supplementary Fig. 17) and after exposure to 8 Torr H_2O vapor referenced to pristine (freshly thermally activated in vacuum, < 20 mTorr), as a function of time in 8 Torr H_2O vapor. The broad band above ~2500 cm^{-1} corresponds to adsorbed H_2O and the sharp mode at 1886 cm^{-1} to adsorbed NO. (b) Time evolution of the intensities of the ν(NO) in capped HKUST-1 before (black diamonds, continuation of Supplementary Fig. 17) and after (orange triangles) exposure to 8 Torr H_2O. The error bars of the ν(NO) normalized integrated band intensity in panel (b) do not exceed 0.02.

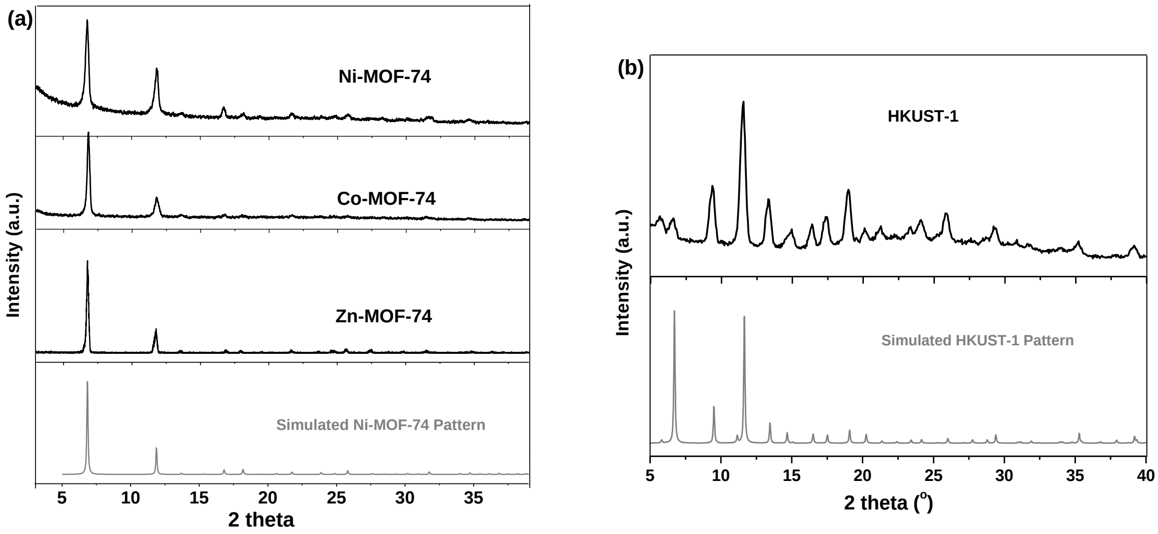

Supplementary Figure 23 / Power X-ray diffraction pattern of (a) Ni, Co, Zn-MOF-74 samples (after solvent exchange) with the simulated pattern from single crystal data from [6]; (b) HKUST-1 sample with the simulated pattern from single crystal data from [7]

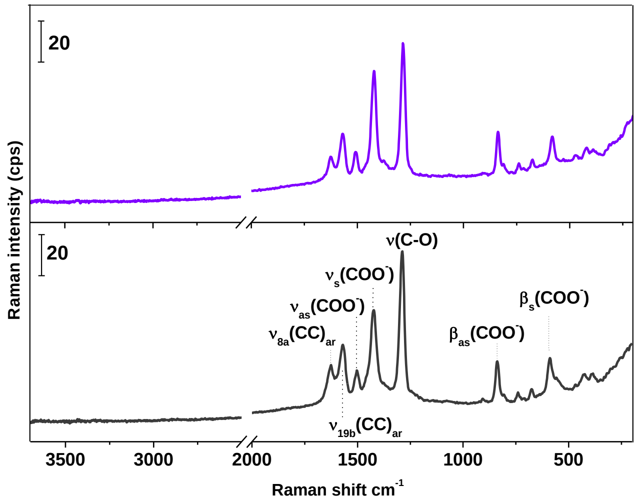

Supplementary Figure 24 / Raman spectra for as-synthesized (bottom panel, black) and post-loaded EDA (top panel, purple) Ni-MOF-74.

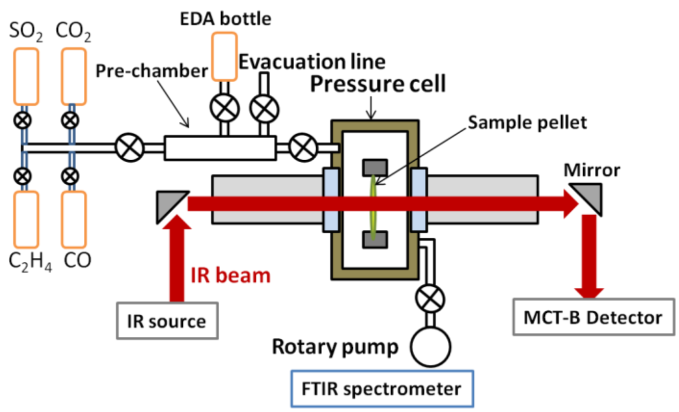

Supplementary Figure 25 / Diagram of the environmental cell placed in the infrared spectrometer.

Supplementary Table 1. Frequencies (cm^{-1}) and binding energies (kJ/mol) of CO molecule co-adsorbed with EDA in Ni-MOF-74 (Supplementary Fig. 4).

	CO alone	CO in state i	CO in state ii	CO in state iii
ν(C-O) frequencies cm^{-1}	2129	2121	2134	2138
Binding energies kJ/mol	51.1	52.4	53.3	49.3

Supplementary Note 1. Post-loading EDA to Ni-MOF-74 with pre-adsorbed CO

After post-loading EDA into Ni-MOF-74 by introducing CO/EDA gas mixture (~40 Torr / ~4 Torr) to the cell over ~10 min, the sample is evacuated by pumping the cell under vacuum (<20 mTorr). The signal of gas-phase CO in the cell disappears quickly (< ~3 second). The remaining peak at 2170 cm^{-1} is due to the adsorbed CO molecules within the MOFs sample (see purple spectrum in Supplementary Fig. 2). EDA bands [$\nu(–NH_2)$, $\nu(–CH_2)$, $\beta(–NH_2)$] are difficult to distinguish because they are in frequency ranges associated with MOF phonon modes (from 1700 to 1000 cm^{-1}) and adsorbed H_2O (> 3000 cm^{-1}) and may also overlap with the gas-phase EDA spectrum, so only the 1020 cm^{-1} peak is shown in Fig. 1 and used for quantitative analyses. The differential spectrum (top), obtained by subtracting the two spectra after evacuating EDA (the top pink spectrum) and before introducing EDA (black), highlights the characteristic bands due to adsorbed EDA molecules within Ni-MOF-74. These involve $\nu(–NH_2)$ at 3300 and 3166 cm^{-1}, $\nu(–CH_2)$ at 2938 and 2880 cm^{-1}, $\beta(–NH_2)$ at 1644, $\nu(C–N)$ at 1020 cm^{-1}. [8] (Supplementary Fig. 2).

Supplementary Note 2. Post-loading NH_3 to Ni-MOF-74 with pre-adsorbed CO

Upon NH_3 loading a previously CO-loaded sample, the intensity of the CO band at 2170 cm^{-1} decreases by ~50% within ~2.5 min and a new band appears at 2136 cm^{-1}, shifted from the adsorbed-phase value 2170 cm^{-1} (Supplementary Fig. 3). This new band is associated with CO molecules displaced by NH_3 from primary adsorption sites on the Ni^{2+} to the secondary sites in the middle of the channel or close to the linker.[9] This is an example of co-adsorption, in which case the molecular frequency of the first species is shifted due to relocation in the unit cell. Clearly, this does not occur for EDA co-adsorption.

Supplementary Note 3. Capping molecules within MOF-74 by other alkyl amine molecules

Besides ethylenediamine, different alkyl amine molecules including trimethylenediamine, n-propylamine, ethanolamine, have been tested to examine the ability of capping small molecules specifically CO within Ni-MOF-74. By the same loading procedure, all these alkyl amine molecules can hinder the desorption of CO from MOF-74 to some extent, however, it was found that EDA is the most effective in retaining CO. This could be due to their propensity to agglomerate as a complete capping layer arising from H-bonding of the head amine groups.

Supplementary Note 4. Diffusion of CO along the one-dimensional channel of Ni-MOF-74

As demonstrated above, the EDA molecules cluster at the periphery of the MOF (the outmost unit cells) in a structure shown in Supplementary Fig. 7(a). We therefore model the diffusion of the CO molecules using two different scenarios: a) all the metal centers saturated with CO molecules (in the absence of EDA cap), and b) all the metal centers saturated with EDA molecules (i.e. through the EDA cap). In both cases, the unit cell contains 6 metal centers, which is a bulk-like environment of the outmost unit cells (i.e. surface effects are neglected). Therefore we believe that this model captures the key elements of the diffusion. To find the lowest energy path, we use a standard transition-state search algorithm, i.e. the nudge elastic band method (NEB), as described in the computational details. In both cases, a CO molecule is placed in the middle of the channel (Fig.2c and Supplementary Fig. 7(b)). Supplementary Figure 7(b) shows that CO penetrates trough the one-dimensional channel of the MOF fully loaded with the same type of molecules by overcoming a diffusion barrier of 0.028 eV, similarly to energy barriers encountered by other small molecules such as CO_2.[10] On the other hand, if the metal centers are now saturated with EDA molecules (scenario depicted in the right panel of Fig. 2c), CO encounters an energy barrier of 0.68 eV (black line of Fig. 2c), i.e. ~24 times larger, due to the larger size of EDA compared to CO. When a CO molecule diffuses through zones where the metal centers are saturated with other CO molecules, they can easily slide though the middle of the one-dimensional channel, barely interacting with the adsorbed CO molecules (see the animation in the supplementary material). In contrast, when a CO molecule diffuses across the channel of the outer pore where EDA molecules are adsorbed on the metal centers, the path is more jagged as CO has to navigate in-between the EDA molecules that are blocking the one-dimensional channel (Supplementary Fig. 9). Moreover, when CO is in close proximity to EDA hydrogen bonding causes the EDA molecules to move and rotate (see the animation in the supplementary material), which greatly increases the energy barrier. This rotation also explains why the red curve in Fig. 2c is not symmetric.

In summary, CO molecule is able to diffuse freely through the one-dimensional channels on a linear trajectory. The situation is very different in the case where the CO molecule tries to diffuse trough the one-dimensional channel of the MOF fully loaded with EDA molecules: the CO molecule has to navigate across the EDA molecule, as illustrated in Supplementary Fig. 9 with the video provided in supplementary information. The interaction between the CO molecule and the EDA molecules causes the large energy barrier. Physically, most CO molecules are reflected back into the MOF.

Supplementary Note 5. Re-loading CO into Ni-MOF-74 with EDA capping on the surface

CO is clearly trapped at room temperature, but can be removed by mild annealing under vacuum (pressure < 20 mTorr). This is shown in the bottom two spectra in Fig. 1 After annealing to 80 °C (~2 h), only ~23% CO remains and the ν(CO) frequency shifts back to 2174 cm^{-1} (no CO–CO interactions); after 100 °C anneal (~2 h), the trapped CO is almost completely removed (~3% remaining; Fig. 1). Importantly after these annealing steps, the spectroscopic signature of EDA molecules, ν(C–N) band at 1020 cm^{-1} remain (with only less than ~30%, intensity decrease), in accordance with the fact that EDA is more strongly bonded to the framework than CO (Fig. 1), as previously observed in MOFs with unsaturated metal centers.[11, 12] The system after annealing is now in a state in which CO has been completely removed yet EDA essentially unperturbed. Therefore, the effect of EDA on CO re-adsorption can now be examined, using the same loading conditions (~40 Torr). Supplementary Figure 10 shows that the CO uptake is dramatically reduced compared to the pristine activated MOF-74 (EDA-free), taking over 45 min to reach only ~25% of the CO loading obtained in pristine MOF-74 loaded in ~30 min. Note that reloading is intrinsically faster than release because trapped molecules are weakly bound to the MOF in contrast to gas-phase molecules.

Supplementary Note 6. Post-loading EDA to Ni-MOF-74 with pre-adsorbed CO_2, SO_2, C_2H_4

The binding energies of CO_2, SO_2, and C_2H_4 in Ni-MOF-74, derived by either isotherm or *ab initio* calculation, are ~38 kJ/mol,[13] ~50–53.5 kJ/mol,[14, 15] and ~42 kJ/mol,[14] respectively, arising mostly from van der Waals and electrostatic interactions. The loading of CO_2, SO_2, and C_2H_4 molecules is performed slightly differently than for CO because the IR absorption of the *gas phase* of these molecules is too strong at ~40 Torr, making IR absorption experiments of *adsorbed gas* impossible (Supplementary Fig. 11–13, a). In order to detect the adsorbed guest molecules during EDA post-loading, the following sequence was followed: preloading Ni-MOF-74 with these molecules (> ~80 Torr), briefly evacuating (~10 sec), then post-loading EDA molecules with ~4 Torr EDA vapor and immediately monitoring the IR absorption (i.e. desorption rates).

For instance, after loading CO_2 into Ni-MOF-74 at ~80 Torr and subsequent evacuation, the CO_2 concentration is estimated at 0.64 CO_2 molecules per metal site.[2] Within ~10 seconds evacuation, the pressure of gas-phase CO_2 drops below ~500 mTorr (negligible gas-phase IR absorption). The adsorbed CO_2 within Ni-MOF-74 is initially detected at 2341 cm^{-1} since its desorption rate is slow (Supplementary Fig. 11a). After introducing ~4 Torr EDA vapor, the IR absorption is recorded for ~10 min during which EDA is kept in the cell. The presence of EDA is confirmed by its $\nu(NH_2)$, $\nu(CH_2)$, and ν(C–N) bands (Supplementary Fig. 11b), and adsorbed CO_2 by its $\nu_{as}(CO_2)$ band at 2341 cm^{-1}. Supplementary Figure S11b shows that, once EDA is adsorbed, the intensity of the CO_2 band at 2341 cm^{-1} stops decreasing. The CO_2 peak center position also does not shift upon loading EDA. Finally, the system is evacuated and the IR absorption spectrum recorded. Monitoring the $\nu_{as}(CO_2$, asymmetric stretch) band of CO_2 molecules adsorbed at the Ni^{2+} site at 2341 cm^{-1},[6] the IR spectrum in Fig. 3a shows that the

$\nu_{as}(CO_2)$ band remains constant (within error bars) in Ni-MOF-74 after 2 h of evacuation. In contrast, the intensity of this band decreases by > ~90% within 20 min in pristine Ni-MOF-74 (Fig. 3a, Supplementary Fig. 14) because CO_2 is weakly bonded to the Ni^{2+} site ($E_{binding}$ = 38 kJ/mol). The stability of CO_2 upon evacuation after post-loading EDA was also measured in Co-MOF-74 and Zn-MOF-74 (Supplementary Fig. 15), suggesting that EDA capping is effective for all metal centers.

After loading SO_2 molecules into Ni-MOF-74 at ~250 Torr for 30 min and subsequent evacuation, two major peaks appear at 1314 cm^{-1} and 1143 cm^{-1}. These are due to antisymmetric ν_{as} and symmetric ν_s bands of physically adsorbed SO_2 molecules (Supplementary Fig. 12a).[16] These two bands fall into the phonon mode (ν_s(COO), ν(CO), β(CH), etc) region of MOF's skeleton,[1] which are perturbed due to SO_2 inclusion into MOFs structure and produce derivative spectroscopic features around ~1200 to ~1100 cm^{-1} (Supplementary Fig. 12b and Fig. 14).[1, 16] After loading EDA into the Ni-MOF-74 at ~4 Torr, the $\nu_{as}(SO_2)$ and $\nu_s(SO_2)$ peaks do not shift or decrease as shown in Supplementary Fig. 12b. During pumping (Fig. 3c), the concentration of adsorbed SO_2 also remains constant in the spectra. This is in stark contrast to the fast decay of these bands in pristine sample (Fig. 3b and Supplementary Fig. 14). The SO_2 concentration within MOFs is recorded by measuring integrated areas of the $\nu_{as}(SO_2)$ peak instead of $\nu_s(SO_2)$ since the asymmetric band $\nu_{as}(SO_2)$ is less affected by β(CH) mode perturbation. There is no literature report for the isotherm data of SO_2 adsorption in Ni-MOF-74. According to previous measurement of SO_2 in Mg-MOF-74,[16] the occupation at ~250 Torr is slightly above ~0.9 molecules per Mg^{2+} site at 297 K.

C_2H_4 adsorption in Ni-MOF-74 is also reversible at room temperature.[17] The most-distinct band was observed at 977 cm^{-1} after loading C_2H_4 at ~200 Torr (Supplementary Fig. 13a) and evacuating gas phase, which was attributed to the $\delta(CH_2$, wagering) mode.[17] There is no literature report for the isotherm data of C_2H_4 adsorption in Ni-MOF-74. According to Bohme's measurement, the occupation of C_2H_4 in both Mg, Co-MOF-74 reaches ~0.6 molecules per metal site at 295 K.[18] Upon evacuation under vacuum, the absorption bands gradually drops, by over 90 % within 2 h. (see red curve of Fig. 3c and spectra of Supplementary Fig. 14). After post-loading EDA at ~4 Torr, the mode ν(C-N) for EDA appears at ~1020 cm^{-1}, close to $\delta(CH_2)$ wagging mode at 977 cm^{-1} (Supplementary Fig. 13b). However, this $\delta(CH_2)$ mode itself is not affected significantly after EDA exposure except for a shift to 970 cm^{-1}. This shift of ~7 cm^{-1} could be due to the interaction of C_2H_4 molecules with trace amount of H_2O impurities adsorbed into MOFs during loading EDA. After pumping for >10 min, it shifts back to the original position at 977 cm^{-1} due to the removal of these water species (Supplementary Fig. 13c). The intensity of $\delta(CH_2)$ mode remains exceptionally stable during subsequent evacuation, which is in stark contrast to the fast decay of these bands in pristine sample (Fig. 3c and Supplementary Fig. 14)

All the trapped CO_2, SO_2, C_2H_4 molecules can be removed by mild annealing (>80 °C) as shown in Supplementary Fig. 11c, 12c, and 13c, so that reloading experiments can be performed. The results are all similar to those for CO: the uptake is lower after EDA exposure and mild

annealing (Supplementary Fig. 11d, 12d, and 13d). These findings suggest that in all cases EDA molecules act as a cap that prevents molecule release from and re-adsorption into MOF-74.

Supplementary Note 7. Interaction between water and EDA molecules within MOFs

As indicated in Fig. 5, there are 8 adsorption sites for a water molecule in the vicinity of the EDA layer. There are therefore 28 ways to add 2 H_2O molecules, 70 ways to add 4 H_2O molecules, and 28 ways to add 6 H_2O molecules. Given all these possible configurations, the resulting areas of the black and red triangles for all those configurations are shown in Supplementary Fig. 19. It is clear that, while some configurations do not increase the triangle areas, most configurations do increase them to a large extend (more than twice in some cases). Similarly for the binding energies given in Supplementary Fig. 20: calculations are performed for each possible configuration, clearly showing that the presence of water decreases the binding energy substantially.

Supplementary Note 8. Raman spectra for as-synthesized and EDA post-loaded Ni-MOF-74 samples

Raman spectra of both as-synthesized sample after solvent exchange and sample with post-loaded EDA were collected by using a Nicolet Almega XR Dispersive Raman spectrometer from Thermo Fisher Scientific, Inc. A 780 nm laser was used for excitation, the output power was reduced to 10% (0.41 mW) to avoid sample decomposition induced by laser heating. As shown in Supplementary Fig. 24, The spectra are dominated by the bulk MOF phonon modes of the carboxylate, phenolate, and aromatic rings, such as $\nu_{as,s}(COO^{-})$, $\beta_{as,s}(COO^{-})$ ν(C–O), and $\nu(C{=}C)_{aromatic\ ring}$, as we have assigned before.[1] The spectroscopic features for EDA modes such as $\nu(NH_2)$ above ~3000 cm^{-1}, $\nu(CH_2)$ between ~3000 and ~2800 cm^{-1}, ν(C-N) between ~1100 and ~900 cm^{-1},[19] ν(Ni-N) in the between ~400 and ~230 cm^{-1} region,[20] cannot clearly be detected in Raman spectrum. This suggests that the amount of EDA adsorption is minimal, only located at the periphery (surface region) of the MOF microcrystals. Furthermore, no notable changes in the spectrum upon post-loading EDA, confirm that the MOF crystalline structure is maintained.

Supplementary Methods

Materials preparation

The MOFs samples are synthesized by following the modified procedure from Ref [3, 13]. Anhydrous redistilled ethylenediamine (EDA, $NH_2(CH)_2NH_2$, ≥ 99.5%) was purchased from Sigma-Aldrich.

Ni-MOF-74: A mixture of nickel nitrate hexahydrate (0.24 g, 0.8 mmol), 2, 5-dihydroxyterephthalic (0.08 g, 0.4 mmol), 9 ml DMF and 1 ml H_2O was prepared in a 28 ml Teflon-lined autoclave. The autoclave was then sealed and heated to 100 ˚C for 3 days. After filtering and washing with 20 ml DMF, the product was collected and exchanged with methanol every 2 h during daytime for at least 3 days. Then the MOFs sample was stored in a N_2 glove box.

Co-MOF-74: A mixture of cobalt nitrate hexahydrate (0.17 g, 0.6 mmol), 2, 5-dihydroxyterephthalic (0.06 g, 0.3 mmol), 9 ml DMF and 1 ml H_2O was prepared in a 28 ml Teflon-lined autoclave. The autoclave was then sealed and heated to 100 ˚C for 3 days. After filtering and washing with 20 ml DMF, the product was collected and exchanged with methanol every 2 h during daytime for at least 3 days. Then the MOFs sample was stored in a N_2 glove box.

Zn-MOF-74: A mixture of zinc nitrate hexahydrate (0.24 g, 0.8 mmol), 2, 5-dihydroxyterephthalic (0.08 g, 0.4 mmol), 9 ml DMF and 1 ml H_2O were transferred into a 28 ml Teflon-lined autoclave. The autoclave was then sealed and heated to 120 ˚C for 3 days. After filtering and washing with 20 ml DMF, the product was collected. Then the product was exchanged with 20 ml methanol in a glass vial every 2 h during daytime for at least 3 days. Then the MOFs sample was stored in a N_2 glove box.

HKUST-1: A solution of copper nitrate hydrate (0.725 g) in deionized water (12 mL) was added to a solution of 1,3,5-benzenetricarboxylic acid (0.42 g) in deionized water (12 mL) and ethyl alcohol absolute (24 mL). The mixture was stirred for 10 min and transferred to a 100 mL Teflon-lined autoclave. The oven was heated at 110 °C for 24 h. The resultant blue crystals were filtered, washed with ethanol and chloroform. Then the MOFs sample was stored in a N_2 glove box.

The crystal structures of MOF samples (Ni, Co, Zn-MOF-74; HKUST-1) were measured by PXRD as shown in Supplementary Fig. 23. The XRD diffraction patterns of the samples we studied are in agreement with literature reports.[6, 7] After thorough solvent exchange, the surface areas reach 913, 1077, 774 m^2/g for Ni-MOF-74, Co-MOF-74, Zn-MOF-74,[1] respectively, consistent with the values reported in the original literature.[13]

Supplementary References:

1. Tan K, *et al.* Water Reaction Mechanism in Metal Organic Frameworks with Coordinatively Unsaturated Metal Ions: MOF-74. *Chem Mater* **26**, 6886-6895 (2014).

2. Yazaydın AOzr, *et al.* Screening of Metal−Organic Frameworks for Carbon Dioxide Capture from Flue Gas Using a Combined Experimental and Modeling Approach. *J Am Chem Soc* **131**, 18198-18199 (2009).

3. Chui SS-Y, Lo SM-F, Charmant JPH, Orpen AG, Williams ID. A Chemically Functionalizable Nanoporous Material [Cu3(TMA)2(H2O)3]n. *Science* **283**, 1148-1150 (1999).

4. Min Wang Q, *et al.* Metallo-organic molecular sieve for gas separation and purification. *Microporous Mesoporous Mater* **55**, 217-230 (2002).

5. Xiao B, *et al.* High-Capacity Hydrogen and Nitric Oxide Adsorption and Storage in a Metal−Organic Framework. *J Am Chem Soc* **129**, 1203-1209 (2007).

6. Dietzel PDC, *et al.* Adsorption Properties and Structure of CO_2 Adsorbed on Open Coordination Sites of Metal-Organic Framework Ni_2(dhtp) from Gas adsorption, IR spectroscopy and X-ray diffraction. *Chem Commun*, 5125-5127 (2008).

7. Xiang S, Zhou W, Gallegos JM, Liu Y, Chen B. Exceptionally High Acetylene Uptake in a Microporous Metal−Organic Framework with Open Metal Sites. *J Am Chem Soc* **131**, 12415-12419 (2009).

8. Chopra TP, Longo RC, Cho K, Halls MD, Thissen P, Chabal YJ. Ethylenediamine Grafting on Oxide-Free H-, 1/3 ML F-, and Cl-Terminated Si(111) Surfaces. *Chem Mater*, (2015).

9. Tan K, *et al.* Competitive Coadsorption of CO_2 with H_2O, NH_3, SO_2, NO, NO_2, N_2, O_2, and CH_4 in M-MOF-74 (M = Mg, Co, Ni): The Role of Hydrogen Bonding. *Chem Mater* **27**, 2203-2217 (2015).

10. Canepa P, Nijem N, Chabal YJ, Thonhauser T. Diffusion of Small Molecules in Metal Organic Framework Materials. *Phys Rev Lett* **110**, 026102 (2013).

11. Hwang YK, *et al.* Amine Grafting on Coordinatively Unsaturated Metal Centers of MOFs: Consequences for Catalysis and Metal Encapsulation. *Angew Chem Int Ed* **47**, 4144-4148 (2008).

12. Choi S, Watanabe T, Bae T-H, Sholl DS, Jones CW. Modification of the Mg/DOBDC MOF with Amines to Enhance CO_2 Adsorption from Ultradilute Gases. *J Phys Chem Lett* **3**, 1136-1141 (2012).

13. Caskey SR, Wong-Foy AG, Matzger AJ. Dramatic Tuning of Carbon Dioxide Uptake via Metal Substitution in a Coordination Polymer with Cylindrical Pores. *J Am Chem Soc* **130**, 10870-10871 (2008).

14. Lee K, Howe JD, Lin L-C, Smit B, Neaton JB. Small-Molecule Adsorption in Open-Site Metal–Organic Frameworks: A Systematic Density Functional Theory Study for Rational Design. *Chem Mater* **27**, 668-678 (2015).

15. Ding L, Yazaydin AÖ. How Well Do Metal–Organic Frameworks Tolerate Flue Gas Impurities? *J Phys Chem C* **116**, 22987-22991 (2012).

16. Tan K, *et al.* Mechanism of Preferential Adsorption of SO2 into Two Microporous Paddle Wheel Frameworks M(bdc)(ted)0.5. *Chem Mater* **25**, 4653-4662 (2013).

17. Chavan S, *et al.* Response of CPO-27-Ni towards CO, N_2 and C_2H_4. *PCCP* **11**, 9811-9822 (2009).

18. Böhme U, *et al.* Ethene/Ethane and Propene/Propane Separation via the Olefin and Paraffin Selective Metal–Organic Framework Adsorbents CPO-27 and ZIF-8. *Langmuir* **29**, 8592-8600 (2013).

19. Krishnan K, Plane RA. Raman and Infrared Spectra of Complexes of Ethylenediamine with Zinc(II), Cadmium(II), and Mercury(II). *Inorg Chem* **5**, 852-857 (1966).

20. Nakamoto K. *Infrared and Raman Spectra of Inorganic and Coordination Compounds, 6th ed Wiley & Sons, Inc,Hoboken, New Jersey, United States*, (**2009**).
