# Supplementary figures and images for: Trapping gases in metal-organic frameworks with a selective surface molecular barrier layer
Source: Nat Commun. 2016 Dec 13;7:13871. doi: 10.1038/ncomms13871 (PMC5159845; doi:10.1038/ncomms13871)

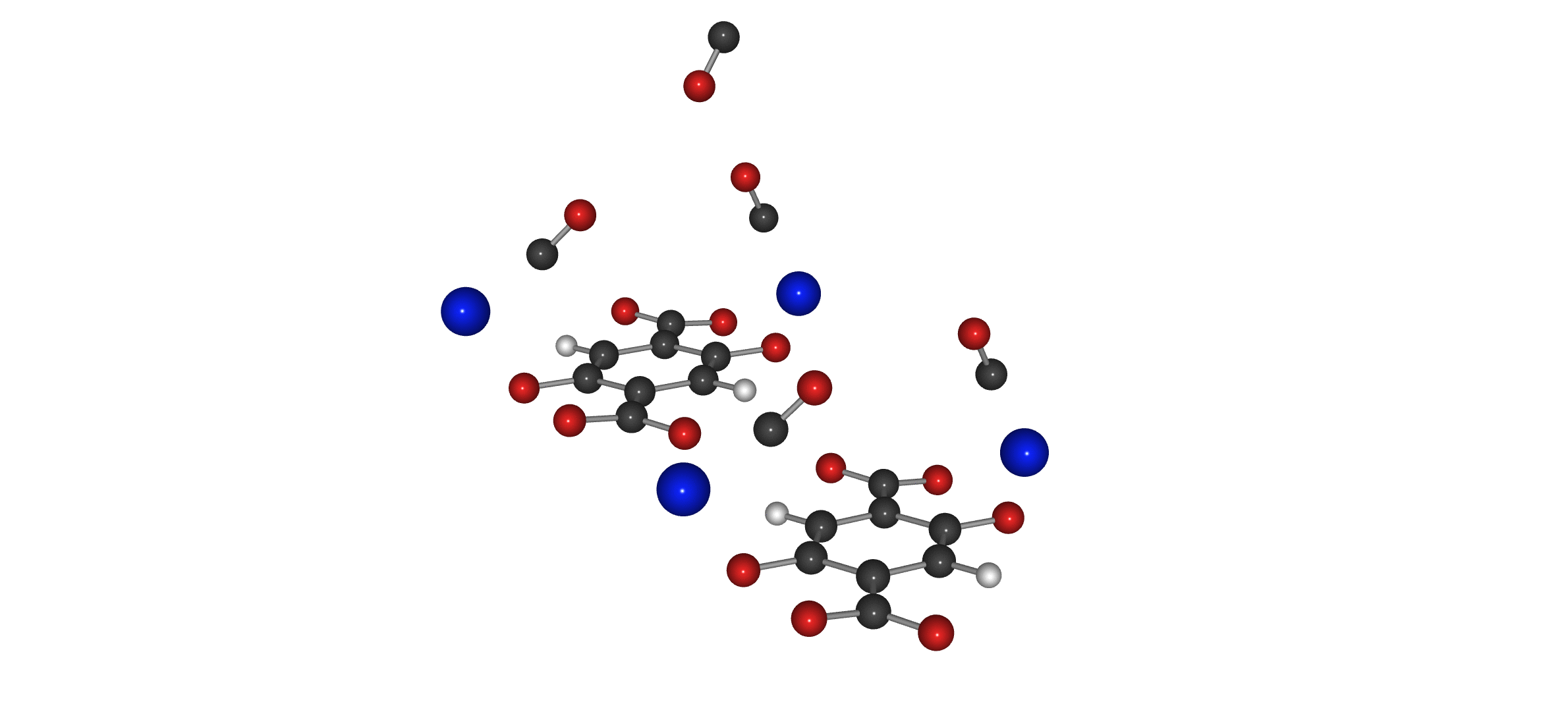

Supplement: Supplementary Movie 1 — CO molecule diffusing through Ni-MOF-74 channel where the metal centers are saturated with other CO molecules [file ncomms13871-s2.gif]

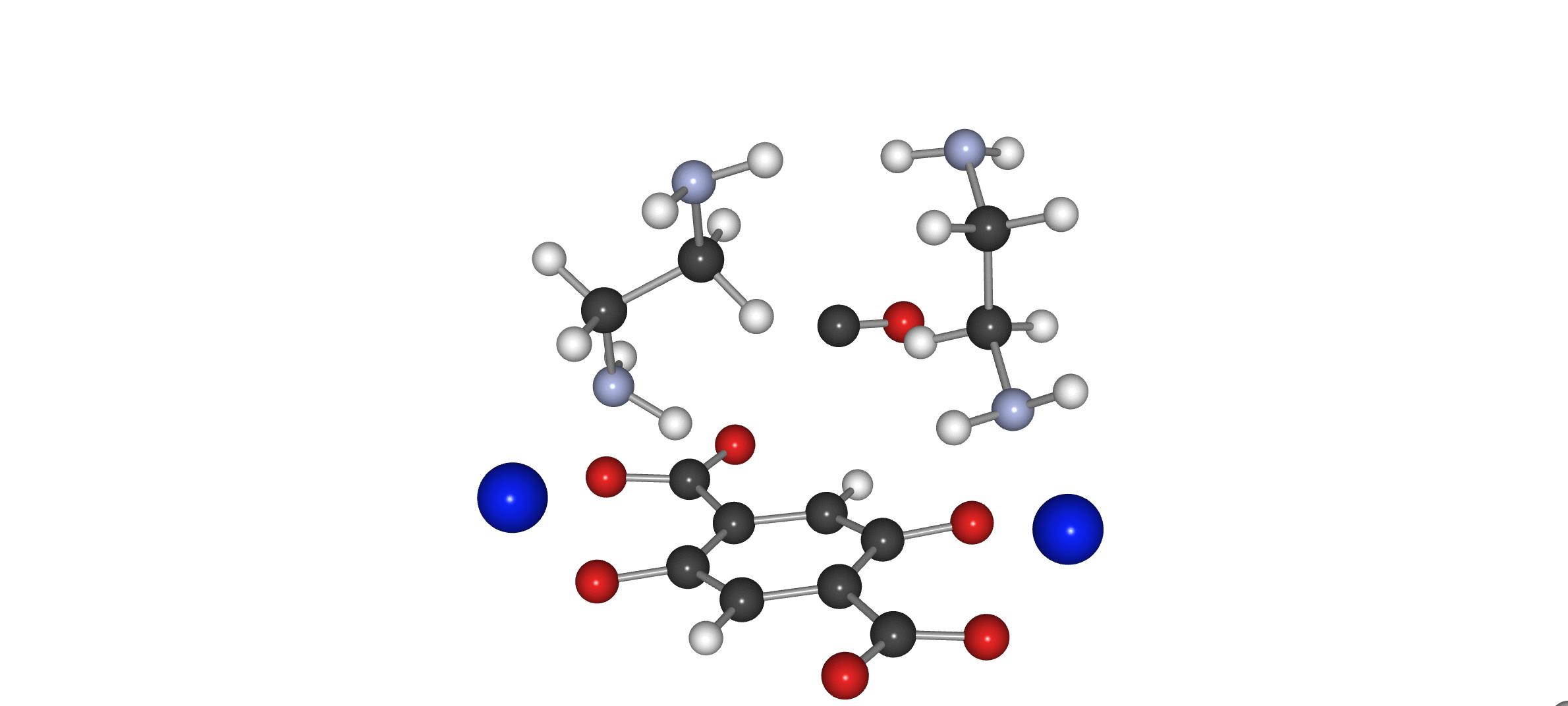

Supplement: Supplementary Movie 2 — CO molecule diffusing through Ni-MOF-74 channel where the metal centers are saturated with ethylenediamine (EDA) molecules [file ncomms13871-s3.gif]
