# Supplementary material for: Trapping gases in metal-organic frameworks with a selective surface molecular barrier layer
Source: Nat Commun. 2016 Dec 13;7:13871. doi: 10.1038/ncomms13871 (PMC5159845; doi:10.1038/ncomms13871)
Supplement: Peer Review File [file ncomms13871-s4.pdf]

Editorial Note: this manuscript has been previously reviewed at another journal that is not operating a transparent peer review scheme. This document only contains reviewer comments and rebuttal letters for versions considered at Nature Communications.

REVIEWERS' COMMENTS:

Reviewer #1 (Remarks to the Author):

I feel that the authors have made a reasonable effort to meet my concerns and also those of the other reviewers, and support publication of this paper as in in Nat Comm. While understanding the reservations of my colleages concerning generality, absolute uniqueness etc, I feel that we do need to respect the conceptual advance of small molecule capping in the sense the authors define it, and that they have done of plenty of work to claim an initial publication. It is increasingly common these days for rather lengthy revision requests to be made regardless of real-life issues such as resource availability, cost-benefit to the extra effort required etc. So I recommend acceptance of the paper in its present form.

Reviewer #2 (Remarks to the Author):

The referees have thoughtfully responded to my previous comments, and I think this article is suitable for publication in Nature Communications.
